# A mathematical investigation into the uptake kinetics of nanoparticles *in vitro*

**DOI:** 10.1371/journal.pone.0254208

**Published:** 2021-07-22

**Authors:** Hannah West, Fiona Roberts, Paul Sweeney, Simon Walker-Samuel, Joseph Leedale, Helen Colley, Craig Murdoch, Rebecca J. Shipley, Steven Webb

**Affiliations:** 1 Mechanical Engineering, University College London, London, United Kingdom; 2 Department for Applied Mathematics, University of Strathclyde, Glasgow, United Kingdom; 3 Cancer Research UK Cambridge Institue, University of Cambridge, Cambridge, United Kingdom; 4 Centre for Advanced Biomedical Imaging, University College London, London, United Kingdom; 5 Department of Mathematical Sciences, University of Liverpool, Liverpool, United Kingdom; 6 School of Clinical Dentistry, University of Sheffield, Sheffield, United Kingdom; 7 Department for Applied Mathematics, Liverpool John Moores University, Liverpool, United Kingdom; 8 Jealott’s Hill, Syngenta, Bracknell, United Kingdom; Polytechnic University of Bucharest, ROMANIA

## Abstract

Nanoparticles have the potential to increase the efficacy of anticancer drugs whilst reducing off-target side effects. However, there remain uncertainties regarding the cellular uptake kinetics of nanoparticles which could have implications for nanoparticle design and delivery. Polymersomes are nanoparticle candidates for cancer therapy which encapsulate chemotherapy drugs. Here we develop a mathematical model to simulate the uptake of polymersomes via endocytosis, a process by which polymersomes bind to the cell surface before becoming internalised by the cell where they then break down, releasing their contents which could include chemotherapy drugs. We focus on two *in vitro* configurations relevant to the testing and development of cancer therapies: a well-mixed culture model and a tumour spheroid setup. Our mathematical model of the well-mixed culture model comprises a set of coupled ordinary differential equations for the unbound and bound polymersomes and associated binding dynamics. Using a singular perturbation analysis we identify an optimal number of ligands on the polymersome surface which maximises internalised polymersomes and thus intracellular chemotherapy drug concentration. In our mathematical model of the spheroid, a multiphase system of partial differential equations is developed to describe the spatial and temporal distribution of bound and unbound polymersomes via advection and diffusion, alongside oxygen, tumour growth, cell proliferation and viability. Consistent with experimental observations, the model predicts the evolution of oxygen gradients leading to a necrotic core. We investigate the impact of two different internalisation functions on spheroid growth, a constant and a bond dependent function. It was found that the constant function yields faster uptake and therefore chemotherapy delivery. We also show how various parameters, such as spheroid permeability, lead to travelling wave or steady-state solutions.

## 1 Introduction

Over one quarter of cancer patients undergo chemotherapy [1]. Unfortunately, the systemic nature of drug delivery and lack of biological specificity of chemotherapeutic agents can result in severe side effects, thus reducing the dose that can be administered [2]. However, in recent years nanoparticle-mediated drug delivery has been developed to target cancer cells, and has shown potential in reducing side effects, whilst increasing intratumoural drug concentration in comparison to traditional chemotherapy [3].

Numerous nanoparticle formations have been investigated for use with anticancer drugs [3]; here we focus on polymersomes which have been shown to have therapeutic potential for head and neck squamous cell carcinoma (HNSCC) patients, among others [4]. HNSCC has extremely poor prognosis outcomes, partly because the chemotherapy dose required to treat the cancer results in significant toxicity to the patient. Therefore new treatment strategies are desperately needed which target the cancer cells and minimise damage to the rest of the body. We previously found that polymersomes bind to and are preferentially taken up by HNSCC cells via class B scavenger receptors that are exposed on their cell surface [5].

**Fig 1:**
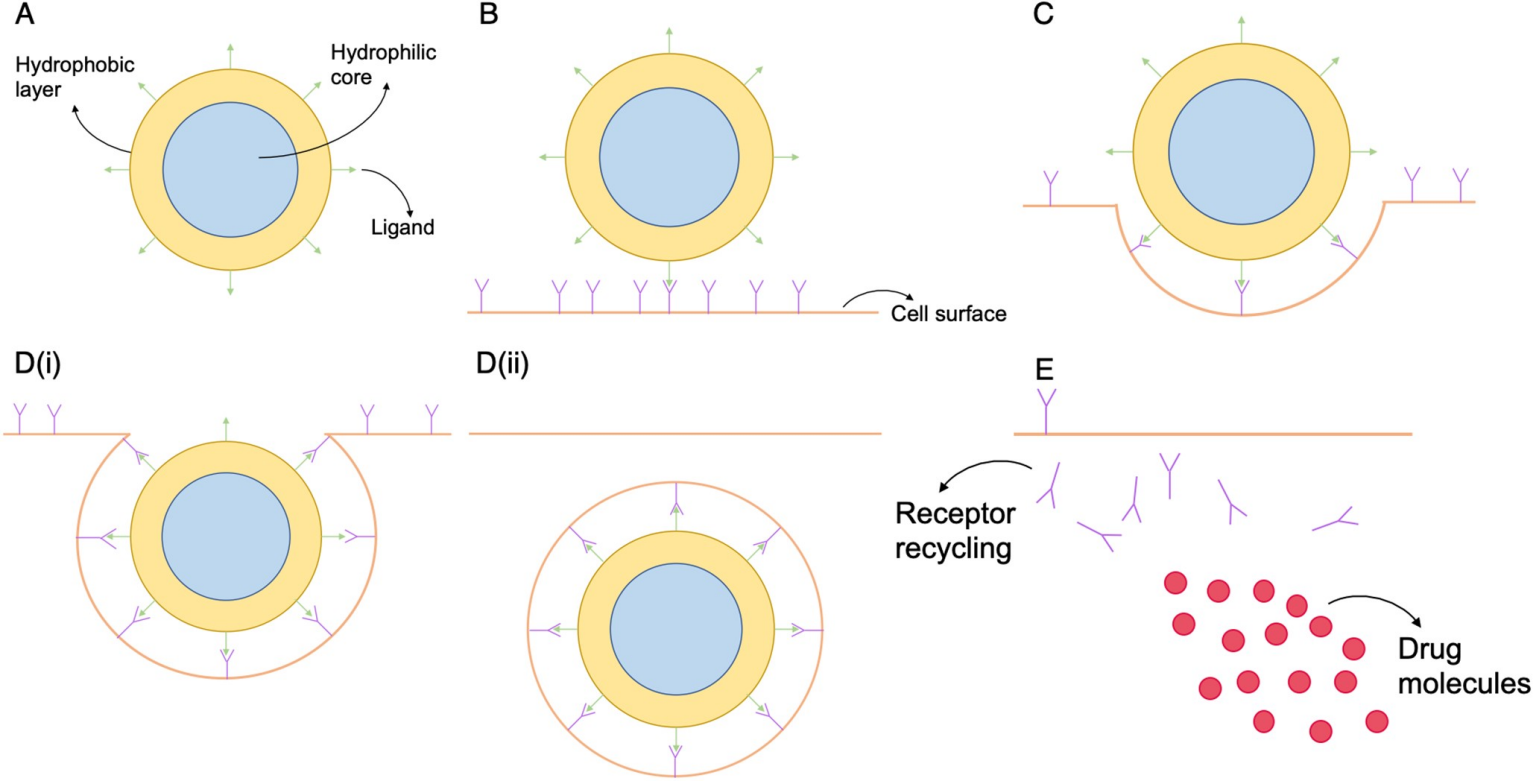
A schematic of the ligand-receptor binding process and internalisation via receptor-mediated endocytosis of polymersomes. **(A)** The structure of polymersomes. **(B)** Receptor-ligand binding, cell surface receptors bind with ligands on the polymersome surface. **(C)** Cell membrane deformation **(D)** The polymersome becomes enclosed by the cell membrane, which breaks away to form an endosome. **(E)** The polymersome ruptures due to the acidic environment of the endosome and releases its contents. Changes in the osmotic pressure with the endosome causes it to rupture, releasing the polymersomes encapsulated chemotherapy drug into the cell. The cell surface receptors that were encapsulated with the polymersome are recycled to the cell surface.

Polymersomes are pH-sensitive synthetic diblock copolymers that self assemble into nanometre-sized vesicles at a neutral pH and disassemble at an acidic pH (below 6.4) [6]. During assembly the polymersomes can encapsulate compounds, such as anticancer drugs, within their core [5, 7]. They are taken up by cells via receptor-mediated endocytosis [8], a process by which the nanoparticles bind to receptors on the cell surface. Once sufficient bonds have formed between the polymersome and cell surface receptor, the polymersome is internalised, becoming surrounded by the plasma membrane that forms an endosome. Endosomal pH is highly acidic which causes the nanoparticle to rapidly disassemble. In turn this causes the endosome to rupture, releasing the anti-cancer drug into the cell cytosol [6]. This process is visualised in Fig 1.

At present, there are still many unknowns around the factors that determine the kinetics of nanoparticle binding, uptake and subsequent chemotherapy release. A more complete understanding of these kinetics will help to enable the optimisation of nanoparticle design and treatment strategies. Here we use mathematical and computational modelling as a complementary tool to existing experimental studies (for example see Murdoch et al [9]) to explore the mechanisms which underpin the process of binding, internalisation and drug release in order to elucidate optimal polymersome design. Computational modelling of chemotherapy delivery to, and distribution within, tumours is a large field of research [10–14].

Previously we developed a mathematical model to describe polymersome uptake via receptor-mediated endocytosis (RME) in a well-mixed culture model [15]. We parameterised the model using *in vitro* experiments and subsequently performed an investigation into the parameters that affect nanoparticle uptake by HNSCC cells. In this study we apply the model to two systems that mimic *in vitro* experimental setups often used in the preclinical stages of drug development. Firstly, we model polymersome uptake in a spatially invariant system that mimics cells cultured in 2D monolayers and focus on key uptake parameters and behaviours when there is abundant polymersome supply. We nondimensionalise the model and perform a singular perturbation analysis to find analytical solutions for the fast binding kinetics of the system. This provides insights into key system behaviours, including the number of binding events that take place before internalisation, which is important because this affects uptake rates, as well as the optimal number of ligands on the particle surface. In an extension to our previous model we also include cells, including their proliferation and death, which impacts uptake, and therefore concentrations of free nanoparticles.

It is important to extrapolate *in vitro* parameters to more physically realistic systems. Therefore we also apply our model to 3D tumour spheroids as they are a more realistic representation of *in vivo* tumour tissue [16] than typical 2D monolayer cultures, yet are a controllable *in vitro* model in which to include the effects of spatial variations in treatment. Due to diffusion limitations of oxygen, tumour spheroids form a necrotic core with a viable outer rim of proliferating cells. This behaviour reflects *in vivo* conditions where heterogeneous vasculature in solid tumours results in hypoxic and ultimately necrotic intra-tumour regions forming. Furthermore, when delivering polymersomes to the spheroid surface we can gain insights into how cells will be impacted at varying distances from a polymersome source, such as a blood vessel. Using spheroids to investigate drug delivery is a common experimental approach for investigating chemotherapy drug delivery [5], as well as a common area of research in computational modelling [17–19].

We use a multiphase model to investigate the effects of various parameters on spheroid growth and we investigate the long term growth of spheroids by exploring travelling wave and steady state behaviours for the tumour boundary. Despite the wealth of literature, as far as we are aware, there are no models published that account for polymersome uptake in a spheroid model which accounts for cell surface receptor recycling and internalisation rates that are polymersome-cell bond dependent, as well as time dependent, which our model is. The goal of our investigation is to identify controllable parameters that could be fed into experimental design of polymersomes in the future.

In the next section, we present the mathematical formulation of our well-mixed system, followed by parameterisation and nondimensionalisation of the model and then a perturbation analysis of the early time periods in the model. Next we present the spheroid model formulation, again followed by parameterisation and nondimensionalisation. We develop numerical solutions for both the well-mixed and spheroid systems, and use them to determine the maximum number of bound and internalised polymersomes as well as the impact of various internalisation functions, all of which play a key role in determining the amount of chemotherapy drug delivered to the tumour cells.

## 2 Well-mixed model

**Fig 2:**
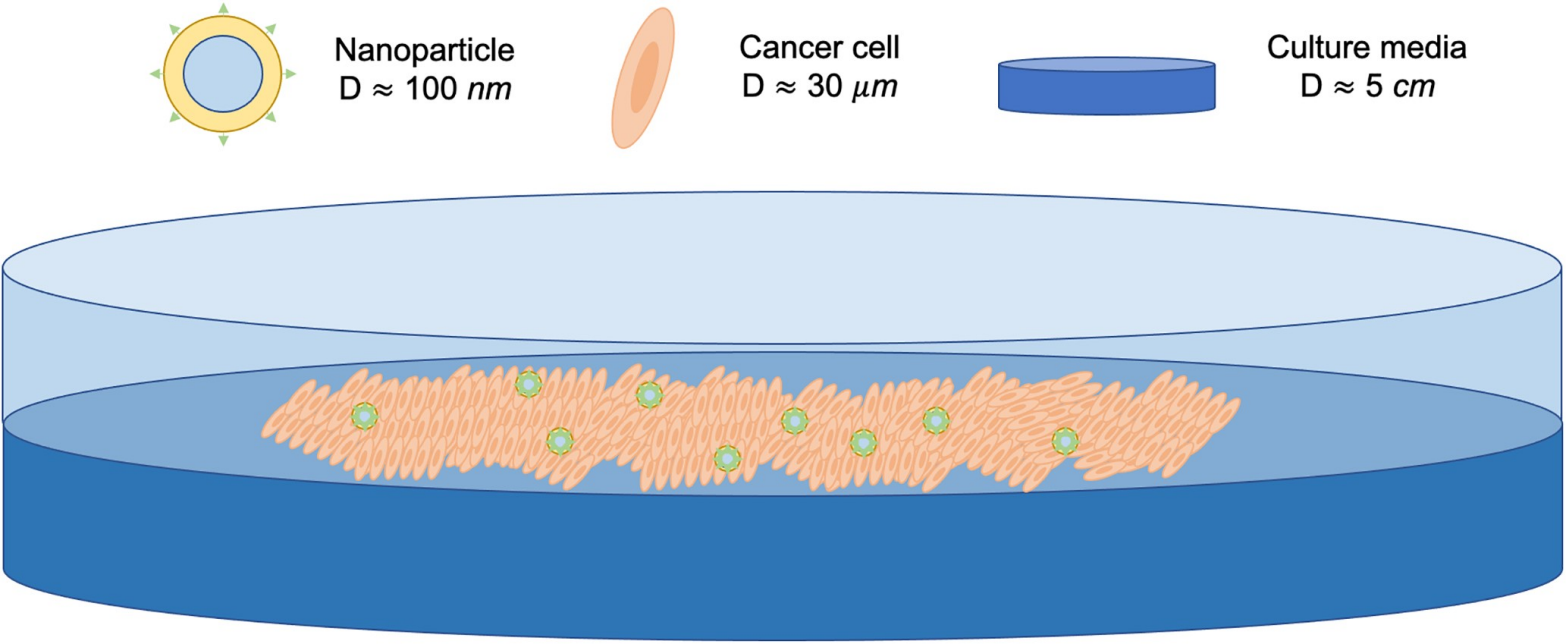
A schematic of the experimental setup of cancer cells and polymersomes in a 2D monolayer cell culture. Cells and seeded in the dish and grown until they have reached optimal capacity. Then therapy is added in order to determine the treatment dosing, mechanisms of action, effectiveness compared to established drugs and interactions with other drugs in a biological environment. The schematic shows the addition of polymersomes to the 2D monolayers. *D* = diameter.

Our first step in cancer drug treatment development with real tissue was to adminster the drug to 2D cell monolayers. This is in order to determine dosing, mechanisms of action, effectiveness compared with established drugs and interactions with other drugs. Here, the polymersomes are assumed to be well-distributed throughout the system, and so we treat this as a spatially invariant system. A simple schematic of this experimental set up is shown in Fig 2.

This is a very common and simple experimental set up, used not just in cancer research but in many applications for other diseases. Mathematical modelling can be used to aid experiments since a large parameter space can be explored quickly. For example, here the number of ligands on the polymersome surface can be quickly varied in order to determine the optimal number for effective treatment. Once key parameters have been identified through modelling these can then be used in experimental systems, thus reducing the time required for these experiments.

### 2.1 Mathematical formulation

The mathematical framework for polymersome uptake in a well-mixed culture model presented here closely follows that of our previously published model. Here we extend the model [15] to include time-varying cell concentration, which we previously assumed to be constant. We use a system of *n*+ 5 ordinary differential equations to describe the uptake of polymersomes (see Fig 1), where *n* is the maximum number of bonds that can form between the polymersome and receptors on the cell surface. We use the same assumptions in our model set up as we did previously. We assume that the system is well-mixed, i.e cells and particles are evenly distributed in space which is representative of *in vitro* conditions, and so ignore any spatial effects. Furthermore we assume that there are a fixed number of available ligands on the polymersome surface that can bind to free cell surface receptors.

Step 1 of RME is the binding of receptors to ligands (see Fig 1a), and therefore the number of free ligands on a polymersome changes over time due to binding with cell surface receptors. We describe the change in the number of free ligands over time, *t*, by

$$\begin{array}{c} \frac{dL}{dt}=-\;\overset{\begin{array}{c} \text{first}\;\text{binding}\\ \text{event} \end{array}}{\overset{︷}{k_{a}LF}}+\overset{\begin{array}{c} \text{dissociation}\;\text{of}\\ \text{complex} \end{array}}{\overset{︷}{k_{d}B_{1},}} \end{array}$$ (1)

where *L* is the moles of free vesicle ligands per cm^3^, *k*_*a*_ is the rate of receptor-ligand binding per minute and *k*_*d*_ is the dissociation rate per minute, *F* is the moles of cell receptors per cm^3^ and *B*_1_ is the moles of receptor-ligand complexes bound with one bond per cm^3^. Here the first term on the RHS of Eq (1) accounts for binding of a free polymersome to a single cell receptor and the second term accounts for dissociation of that bond such that particles are no longer bound to the cell.

From Eq (1) we can calculate the number of free polymersomes per cm^3^, V, by

$$\begin{array}{c} V(t)=L/l, \end{array}$$ (2)

where *l* is the fixed moles of free ligands per polymersome. We use the law of mass action to describe the binding kinetics so receptor-ligand complexes with one bond change over time through,

$$\begin{array}{ccc} \frac{dB_{1}}{dt} & = & \overset{\begin{array}{c} \text{first}\;\text{binding}\\ \text{event} \end{array}}{\overset{︷}{k_{a}LF}}-\overset{\begin{array}{c} \text{dissociation}\;\text{of}\\ \text{complex} \end{array}}{\overset{︷}{k_{d}B_{1}}}-\;\overset{\begin{array}{c} \text{association}\;\text{of}\\ 1\to 2\;\text{complexes} \end{array}}{\overset{︷}{k_{a}(\rho_{l}lN_{A}-1)\rho_{f}FB_{1}}}\\ & & +\overset{\begin{array}{c} \text{dissociation}\;\text{of}\;2\to 1\\ \text{complexes} \end{array}}{\overset{︷}{2k_{d}B_{2}}}-\overset{\begin{array}{c} \text{internalisation}\;\text{of}\\ \text{complex}\;\text{with}\;\text{1}\;\text{bond} \end{array}}{\overset{︷}{k_{in}B_{1},}} \end{array}$$ (3)

where *N*_*A*_ is Avogadro’s constant, *B*_2_ is the moles of receptor-ligand complexes bound with two bonds per cm^3^, *k*_*in*_ is the polymersome internalisation rate per minute and *ρ*_*l*_ and *ρ*_*f*_ are the fraction of ligands and receptors respectively that are available for 2-D binding, i.e only ligands and receptors within a certain range can form bonds. The term 2-D binding is used to describe the subsequent binding after one initial receptor-ligand bond had formed.

Eq (3) involves terms that are functions of *ρ*_*l*_ and *ρ*_*f*_. In the third term on the RHS of Eq (3), (*ρ*_*l*_ *lN*_*A*_ − 1) represents the ligands available on the polymersome for subsequent binding after the initial binding event. The term describes that there is one less ligand available for subsequent binding, in which the polymersome would become bound to the cell with two bonds (for example see Fig 1B). The fourth term on the RHS represents dissociation of a bond, meaning that a new receptor and ligand are now free binding at a rate 2*k*_*d*_ (where the factor 2 represents that any one of the two bonds can dissociate). Internalisation of polymersomes occurs at a constant rate *k*_*in*_; in this well-mixed model we assume that the binding rate and internalisation rate are independent of the number of bonds that have already formed following our previously published model [15]. Bond dependent internalisation is explored later in this paper.

Generalising, the number of receptor-ligand complexes bound by *i* bonds changes over time by

$$\begin{array}{ccc} \frac{dB_{i}}{dt} & = & \overset{\begin{array}{c} \text{association}\;\text{of}\\ (i-1)\to i\;\text{complexes} \end{array}}{\overset{︷}{k_{a}(\rho_{l}lN_{a}-\left(i-1\right))\rho_{f}FB_{i-1}}}\;\;-\overset{\begin{array}{c} \text{dissociation}\;\text{of}\;\;i\to (i-1)\\ \text{complexes} \end{array}}{\overset{︷}{ik_{d}B_{i}}}\\ & & -\;\overset{\begin{array}{c} \text{association}\;\text{of}\\ i\to (i+1)\;\text{complexes} \end{array}}{\overset{︷}{k_{a}(\rho_{l}lN_{a}-i)\rho_{f}FB_{i}}}\;\;+\overset{\begin{array}{c} \text{dissociation}\;\text{of}\;\;(i+1)\to i\\ \text{complexes} \end{array}}{\overset{︷}{\left(i+1\right)k_{d}B_{i+1}}}\\ & & -\underset{\begin{array}{c} \text{internalisation}\;\text{of}\\ \text{complex}\;\text{with}\;i\;\text{bonds} \end{array}}{\overset{︷}{k_{in}B_{i},}} \end{array}$$ (4)

where 2 ≤ *i* ≤ *n* − 1 and *n* is the maximum number of ligand-receptor bonds that can form.

The number of receptor-ligand complexes bound by *n* bonds change over time by,

$$\begin{array}{ccc} \frac{dB_{n}}{dt} & = & \overset{\begin{array}{c} \text{association}\;\text{of}\\ (n-1)\to n\;\text{complexes} \end{array}}{\overset{︷}{k_{a}(\rho_{l}lN_{a}-\left(n-1\right))\rho_{f}FB_{n-1}}}-\overset{\begin{array}{c} \text{dissociation}\;\text{of}\;\;n\to (n-1)\\ \;\text{complexes} \end{array}}{\overset{︷}{nk_{d}B_{n}}}\\ & & \;-\underset{\begin{array}{c} \text{internalisation}\;\text{of}\\ \text{complex}\;\text{with}\;n\;\text{bonds} \end{array}}{\overset{︷}{k_{in}B_{n}.}} \end{array}$$ (5)

When a maximal number of bonds, *n*, is reached, polymersomes are internalised into endosomes (see Fig 1D.ii). The acidic environment of the endosome causes polymersomes to break down and release the encapsulated drug. The rate of change of internalised polymersomes over time is then given by,

$$\begin{array}{ccc} \frac{dB_{in}}{dt} & = & \overset{\text{total}\;\text{internalised}\;\text{polymersomes}}{\overset{︷}{\frac{k_{in}}{l}\sum_{i}^{n}B_{i}}}-\overset{\text{polymersome}\;\text{breakdown}}{\overset{︷}{d_{b}B_{in}.}} \end{array}$$ (6)

Internalised polymersomes are lost due to polymersome rupture at an assumed constant rate, the rupture rate *d*_*b*_. When polymersomes rupture within the cell they release the encapsulated chemotherapy drug (see Fig 1E), giving the rate of change of intracellular drug as,

$$\begin{array}{ccc} \frac{dP}{dt} & = & \overset{\text{drug}\;\text{released}\;\text{by}\;\text{polymersome}\;\text{breakdown}}{\overset{︷}{\nu d_{b}B_{in}}}-\overset{\text{drug}\;\text{half-life}}{\overset{︷}{d_{p}P,}} \end{array}$$ (7)

where *P* is the concentration of intracellular drug per cm^3^. The release of the drug is proportional to the rupture of the polymersome, *ν*, and we also include a decay in drug activity which we assume occurs at constant rate, *d*_*p*_, which corresponds to uptake or removal of the chemotherapy drug.

During the process of binding there is a change in the number of free receptors, *F*, on the cell surface. This is because once a receptor binds with a ligand it is no longer free to bind to other ligands, and becomes internalised in the process of endocytosis. Once inside the cell, the receptors are recycled back to the cell surface [20] (see Fig 1E). We describe this rate of change of free receptors by,

$$\begin{array}{ccc} \frac{dF}{dt} & = & -\;\overset{\begin{array}{c} \text{initial}\;\text{receptor-}\\ \text{ligand}\;\text{binding} \end{array}}{\overset{︷}{k_{a}FL}}-\overset{\text{subsequent}\;\text{binding}\;\text{with}\;\text{ligands}}{\overset{︷}{k_{a}\sum_{i}^{n}\left(\rho_{l}lN_{a}-i\right)\rho_{f}FB_{i}}}\\ & & \;+\overset{\text{dissociation}\;\text{of}\;i\;\text{bonds}}{\overset{︷}{k_{d}\sum_{i}^{n}iB_{i}}}-\overset{\text{receptor}\;\text{half-life}}{\overset{︷}{d_{f}F}}+\overset{\text{receptor}\;\text{production}}{\overset{︷}{R(b_{tot})m,}} \end{array}$$ (8)

where we have assumed that receptors have a limited life on the cell surface, with the constant linear decay rate *d*_*f*_. We assume that receptor production occurs on the cell surface and that receptors are recruited to the cell surface at a rate proportional to the number of bound receptors. We base these assumptions on the similar model of Ghaghada et al [21] who investigate receptor recycling and nanoparticle-cell bonds through receptor-mediated targeting for liposomes. We describe receptor recycling using a function of the total number of bound receptors, *R*(*b*_*tot*_), where *b*_*tot*_ is the total number of bound complexes per cell. We assume *R*(*b*_*tot*_) is constant if there are no bound receptors (b_*tot* = 0_), otherwise we assume an increasing saturating function dependent on the total number of bound complexes per cell. *R*(*b*_*tot*_) is given by

$$\begin{array}{c} R\left(b_{tot}\right)=c_{1}+\frac{c_{2}b_{tot}^{\alpha }}{c_{3}^{\alpha }+b_{tot}^{\alpha }}\;\text{where}\;b_{tot}=\frac{1}{m}\sum_{i}^{n}iB_{i}, \end{array}$$ (9)

*m* is the number of tumour cells per cm^3^, *α* is the Hill exponent, *c*_1_ is the rate of receptor production and *c*_2_ and *c*_3_ characterise the hill equation. Here *c*_2_ is the maximum recycling rate and *c*_3_ is the value at which the half maximum of the function *R*(*b*_*tot*_) is reached.

In an extension to our previous model we describe how the tumour cell density changes over time due to the balance of proliferation and death through

$$\begin{array}{ccc} \frac{dm}{dt} & = & \overset{\text{growth}}{\overset{︷}{rm(1-\frac{m}{K})}}-\overset{\text{death}}{\overset{︷}{g(\phi )m,}} \end{array}$$ (10)

where *m* is the number of cells per cm^3^, the carrying capacity of the system is *K* and we assume that the cells undergo logistic proliferation at constant rate, *r*. We include cells in our model since the number of cells directly correlates to the uptake amount of nanoparticles and therefore the number of remaining free polymersomes. Cell death is described by the function *g*(*ϕ*), which we assume is constant when there is no intracellular drug present due to natural cell death, otherwise it is an increasing saturating function of the intracellular drug concentration [22]. Cell death is then described through

$$\begin{array}{c} g\left(\phi \right)=d_{m}+\frac{\mu \phi^{\beta }}{P_{0}^{\beta }+\phi^{\beta }}, \end{array}$$ (11)

where $\phi \left(t\right)=\frac{P}{m}$ describes the mole of drug per cell, *P* is the moles of drug per cm^3^, *P*_0_ is the drug concentration that produces a 50% maximal response, *d*_*m*_ is the natural cell death, *β* is the Hill exponenent and *μ* is the cell death rate due to drug delivery. The functions of cell death, *g*(*ϕ*), and receptor recycling, *R*(*b*_*tot*_) are generalised hill equations which are commonly used to model cell kinetics and nutrient consumption, for example see references [22–24].

We further impose the following initial conditions,

$$\begin{array}{cccc} m\left(0\right)=M_{0}, & V\left(0\right)=V_{0}, & & F\left(0\right)=F_{0},\\ B_{i}\left(0\right)=0,\; & B_{in}\left(0\right)=0 & \text{and} & P(0)=0, \end{array}$$

where *i* = 1, …, *n*, *M*_0_ is the initial cell concentration, *V*_0_ is the initial polymersome concentration, *F*_0_ is the initial cell surface receptor concentration, *B*_*i*_(0) is the concentration of polymersomes bound with *i* complexes, *B*_*in*_(0) is the internalised polymersome concentration and *P*(0) is the released drug concentration, all at time *t* = 0. These conditions state that initially there are no bound or internalised polymersomes present when the cells and particles are mixed together and therefore there is no released drug.

### 2.2 Parameterisation

The parameterisation of the system proved difficult since the majority of the parameters for specific polymersome binding are unknown due to a lack of relevant experimental data. Hence we look to a similar system of liposomal targeting which has similar dynamics to those of polymersome targeting and is more well understood experimentally. Specifically we look to use parameters from a liposomal modelling study by Ghaghada *et al*. [21], in which targeted liposomes bind to folate receptors of C6 glioma cells. The parameter values used in the subsequent analysis are shown in Table 1. Further information on how the parameters were chosen is provided in the S1 File.

**Table 1:** Model parameter values. Where no data were available in the literature we estimated parameters values to match experimental observations. See S1 File.

Parameter	Dimensional Value	Dimensionless Values	Reference
Initial cell concentration, *M*_0_	5 × 10^7^ cell cm^−3^	1	[25]
Initial polymersome concentration, *V*_0_	10^10^ polymersome cm^−3^	1	estimate
Initial cell surface receptor concentration, *F*_0_	1.66 × 10^−20^ mol cell^−1^	1	[21]
Half maximal drug concentration, *P*_0_	4.15 × 10^−14^ mol cell^−1^	-	estimate
Cell proliferation rate, *r*	6 × 10^−5^ min^−1^	-	[25]
Tissue carrying capacity, *K*	5 × 10^7^ cells cm^−3^	-	estimate
polymersome binding rate, *k*_*a*_	3.7010 × 10^8^ mol^−1^min^−1^cm^3^	0.4451	[21]
polymersome dissociation rate, *k*_*d*_	3.7010 × 10^−5^ min^−1^	0.0533	[21]
polymersome internalisation rate, *k*_*in*_	0.6124 min^−1^	887.5	[21]
Avogadros constant, *N*_*a*_	6.022 × 10^23^ molecules	-	-
Ligands per polymersome, *l* ⋅ *N*_*a*_	1200 ligands polymersome^−1^	-	estimate
Receptor decay rate, *d*_*f*_	0.03 min^−1^	43.203	[26]
Fraction of receptors available for binding, *ρ*_*f*_	(0, 1]	-	estimate
Fraction of ligands available for binding, *ρ*_*l*_	(0, 1]	-	estimate
polymersome rupture rate, *d*_*b*_	2.3 min^−1^	3.3122	[8]
Drug decay rate, *d*_*p*_	1.2 min^−1^	1739	estimate
Natural cell death rate, *d*_*m*_	0.01min^−1^	724.6	[27]
Cell death due to drug, *μ*	1.2 min^−1^	1739	[8]
Receptor production rate, *c*_1_	4.98 × 10^−22^ mol cell^−1^ min^−1^	43.478	estimate
Maximum receptor recycling rate, *c*_2_	4.98 × 10^−22^ mol cell^−1^ min^−1^	43.478	estimate
Half maximum receptor recycling concentration, *c*_3_	2.76 × 10^−20^mol cell^−1^	83.28	estimate
Hill exponent (receptor recycling), *α*	-	1	estimate
Hill exponent (cell death), *β*	-	1	estimate
Receptor scaling factor, *ν*	-	8.414 × 10^−5^	estimate
Scaling parameter, *η*	-	0.02	estimate

### 2.3 Nondimensionalisation

We non-dimensionalise the model in order to understand the dominant terms in the system and to enable prioritisation of the parameters needed for future experiments. We rescale the system as follows:

$$\begin{array}{llll} m=\hat{m}K, & V=\hat{V}V_{0}, & F=\hat{F}F_{0}KN_{A}, & B_{i}={\hat{B}}_{i}V_{0},\\ B_{in}=\hat{B_{in}}V_{0}, & P=\hat{P}P_{0}K & t=\frac{\hat{t}}{r}, & \end{array}$$

where *i* = 1, …, *n* and the non-dimensional variables are denoted by hats. Removing the hats for notational convenience the rescaled system then reads

$$\begin{array}{ccc} \frac{dm}{dt} & = & m\left(1-m\right)-\tilde{g}\left(\tilde{\phi }\right)m, \end{array}$$ (12)

$$\begin{array}{ccc} \frac{dV}{dt} & = & -{\tilde{k}}_{a}VF+\frac{{\tilde{k}}_{d}B_{1}}{l}, \end{array}$$ (13)

$$\begin{array}{ccc} \frac{dB_{1}}{dt} & = & {\tilde{k}}_{a}LF-{\tilde{k}}_{d}B_{1}-{\tilde{k}}_{in}B_{1}\\ & & -{\tilde{k}}_{a}\left(\rho_{l}l-1\right)\rho_{f}FB_{1}+2{\tilde{k}}_{d}B_{2}, \end{array}$$ (14)

$$\begin{array}{ccc} \frac{dB_{i}}{dt} & = & {\tilde{k}}_{a}\left(\rho_{l}l-\left(i-1\right)\right)\rho_{f}FB_{i-1}-i{\tilde{k}}_{d}B_{i}-{\tilde{k}}_{in}B_{i}\\ & & -{\tilde{k}}_{a}\left(\rho_{l}l-i\right)\rho_{f}FB_{i}+\left(i+1\right){\tilde{k}}_{d}B_{i+1}, \end{array}$$ (15)

$$\begin{array}{ccc} \frac{dB_{n}}{dt} & = & {\tilde{k}}_{a}\left(\rho_{l}l-\left(n-1\right)\right)\rho_{f}FB_{n-1}-{\tilde{k}}_{in}B_{n}-n{\tilde{k}}_{d}B_{n}, \end{array}$$ (16)

$$\begin{array}{ccc} \frac{dF}{dt} & = & -\eta {\tilde{k}}_{a}FL-\eta {\tilde{k}}_{a}\sum_{i}^{n}\left(\rho_{l}l-i\right)\rho_{f}FB_{i}+\eta {\tilde{k}}_{d}\sum_{i}^{n}iB_{i}\\ & & -{\tilde{d}}_{f}F+\tilde{R}\left({\tilde{b}}_{tot}\right)m, \end{array}$$ (17)

$$\begin{array}{ccc} \frac{dB_{in}}{dt} & = & \frac{{\tilde{k}}_{in}}{l}\sum_{i}^{n}B_{i}-{\tilde{d}}_{b}B_{in}, \end{array}$$ (18)

$$\begin{array}{ccc} \frac{dP}{dt} & = & \tilde{\nu }{\tilde{d}}_{b}B_{in}-{\tilde{d}}_{p}P, \end{array}$$ (19)

with

$$\begin{array}{c} \tilde{g}\left(\tilde{\phi }\right)={\tilde{d}}_{m}+\frac{\tilde{\mu }\phi^{\beta }}{P_{0}^{\beta }+\phi^{\beta }}\;\text{and}\;\tilde{R}\left({\tilde{b}}_{tot}\right)={\tilde{c}}_{1}+\frac{{\tilde{c}}_{2}{\tilde{b}}_{tot}^{\alpha }}{{\tilde{c}}_{3}^{\alpha }+{\tilde{b}}_{tot}^{\alpha }}, \end{array}$$ (20)

where

$${\tilde{b}}_{tot}=\frac{1}{m}\sum_{i}^{n}iB_{i}.$$

The rescaled parameters, denoted by tildes, are defined as

$$\begin{array}{lllll} {\tilde{k}}_{a}=\frac{k_{a}f_{0}K}{r}, & {\tilde{k}}_{d}=\frac{k_{d}}{r}, & {\tilde{k}}_{in}=\frac{k_{in}}{r}, & {\tilde{d}}_{m}=\frac{d_{m}}{r}, & \tilde{\mu }=\frac{\mu }{r},\\ \eta =\frac{V_{0}}{N_{A}f_{0}K}, & {\tilde{d}}_{f}=\frac{d_{f}}{r}, & {\tilde{d}}_{b}=\frac{d_{b}}{r}, & {\tilde{d}}_{p}=\frac{d_{p}}{r}, & \tilde{\nu }=\frac{V_{0}\nu }{P_{0}K},\\ {\tilde{c}}_{1}=\frac{c_{1}}{f_{0}r}, & {\tilde{c}}_{2}=\frac{c_{2}}{f_{0}r}, & {\tilde{c}}_{3}=\frac{c_{3}K}{V_{0}}, & \tilde{l}=\frac{l}{N_{A}}. & \end{array}$$

The rescaled system is then subject to the following initial conditions,

$$\begin{array}{c} m\left(0\right)=1,\;V\left(0\right)=1,\;F\left(0\right)=1,\;B_{i}\left(0\right)=0,\;B_{in}\left(0\right)=0,\;P\left(0\right)=0, \end{array}$$ (21)

for *i* = 1, …, *n*. The values of the rescaled parameters are given in Table 1. We see that in the rescaled system the larger parameters (on the order of or close to 10^3^) are the internalisation rate, *k*_*in*_, the polymersome rupture rate, *d*_*b*_, the drug decay rate, *d*_*p*_, the natural cell death rate, *d*_*m*_, and cell death due to drug, *μ*, so we expect that these will have large influences on the system. On the other hand the initial binding rate, *k*_*a*_, and the dissociation rate, *k*_*d*_, are small (less than one) so we expect these to have a negligible impact on the system.

**Fig 3:**
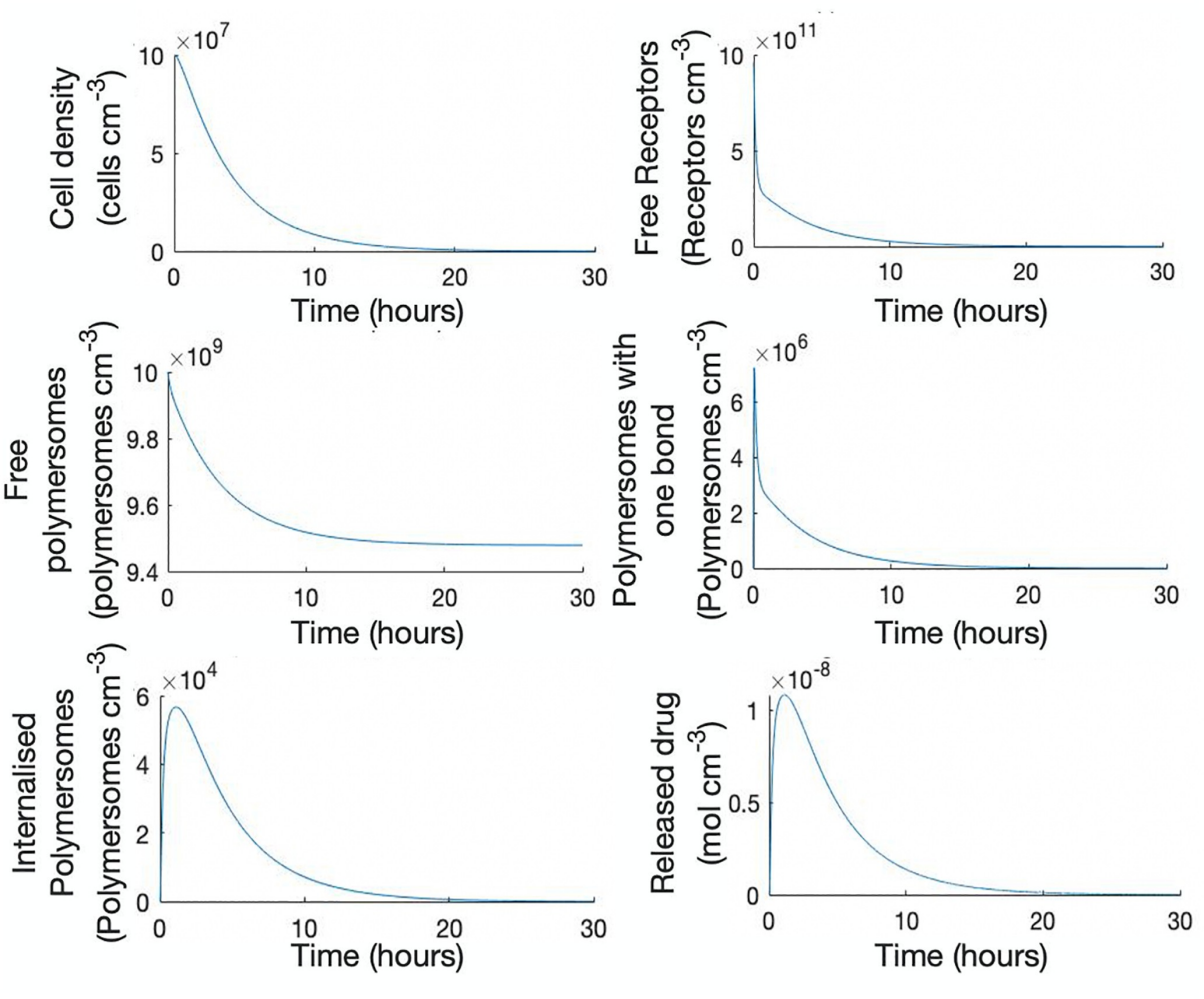
Predictions of the model (Eqs 12–19) for the parameter values given in Table 1. The nondimensional system and parameters were used for the simulations and the results were converted to dimensional values. The maximum number on bons, *n* = 20.

We solve the system numerically using the ODE solver ode45 in Matlab, which is based on the explicit Runge-Kutta 45 method. Fig 3 shows representative results for a typical set of parameter values, given in Table 1. Initially there is a quick decrease in free receptors due to the fast initial binding with ligands on the polymersome surfaces, then a more gradual decline is observed as the number of tumour cells, and hence, cell surface receptors decrease. With the maximum number of complexes being set to twenty, the majority of polymersomes only create a small number of bonds with the cell surface before being internalised, with the maximum number of internalised polymersomes occurring after around one hour. The intracellular drug concentration exhibits a similar behaviour as the internalised polymersomes. This is to be expected as the drug release is assumed to be proportional to the rate of internalisation of polymersomes. The simulations show that, for this specific set of parameter values, the tumour cell population decreases to zero over time after polymersomes are introduced. The decrease is rapid over approximately the first 10 hours and then slows as the population approaches zero.

### 2.4 Singular perturbation analysis

The results of the numerical system show rapid changes in the system at early time points. This is to be expected based on the large coefficients we find in our nondimensionalisation. To get an analytical handle on this we carry out a singular perturbation analysis of early time points in the system. We use the non-dimensional system given in the previous section 2.3 with the hats and tildes dropped for convenience.

For simplicity, we first consider a single binding interaction, where only one complex between a ligand and receptor has to form for the drug-loaded polymersome to be internalised (n = 1). We justify this by observing that the binding rate is much smaller than the internalisation rate so that a polymersome will likely be internalised after one bond has formed and before other bonds form. We do this by setting *ρ*_*l*_ = *l*^−1^ and *ρ*_*f*_ = 0 so that *B*_*i*_(*t*) = 0 for 2 ≤ *i* ≤ *n*.

For further simplification of the model we look at the parameters involved in the kinetics of receptors and ligands. Note that, with the parameter values described in the previous section, we have the dimensionless values, *k*_*d*_ ∼ *O*(10^−2^), $\frac{k_{d}}{l}∼O\left(10^{-5}\right)$ and *ηk*_*d*_ ∼ *O*(10^−4^) and so we set the dissociation rate *k*_*d*_ = 0. Hence we can write $\eta l=\frac{\hat{\eta l}}{\varepsilon }$, $d_{f}=\frac{\hat{d_{f}}}{\varepsilon }$ and $c_{1}=\frac{\hat{c_{1}}}{\varepsilon }$ where *ε* ≪ 1 is a scaling parameter. We also look to simplify the saturating functions for receptor recycling, *R*(*b*_*tot*_), and cell death, *g*(*ϕ*), given by (20). The total cell death rate, *g*(*ϕ*), can be linearised since $P_{0}^{\beta }$ is neglible thus $\tilde{g}\left(\tilde{\phi }\right)={\tilde{d}}_{m}+\frac{\tilde{\mu }\phi^{\beta }}{P_{0}^{\beta }+\phi^{\beta }}$ reduces to $\tilde{g}\left(\tilde{\phi }\right)={\tilde{d}}_{m}+\tilde{\mu }$. To simplify $R({\tilde{b}}_{tot})$, we assume that *c*_3_ is small compared to ${\tilde{b}}_{tot}$, so that *R*(*b*) ∼ *c*_1_ + *c*_2_ ≔ *R*.

Before performing the analysis we make one further assumption in order to simplify the system, assuming *t* = *τε* where *τ* represents short time behaviour. Using these assumptions the system then becomes

$$\begin{array}{ccc} \frac{dm}{d\tau } & = & \varepsilon m(1-m)-\varepsilon g(\phi )P, \end{array}$$ (22)

$$\begin{array}{ccc} \frac{dV}{d\tau } & = & -\varepsilon k_{a}VF, \end{array}$$ (23)

$$\begin{array}{ccc} \frac{dF}{d\tau } & = & -k_{a}\eta lVF-d_{f}F+Rm, \end{array}$$ (24)

$$\begin{array}{ccc} \frac{dB}{d\tau } & = & \varepsilon k_{a}lVF-\varepsilon k_{in}B, \end{array}$$ (25)

$$\begin{array}{ccc} \frac{dB_{in}}{d\tau } & = & \varepsilon \frac{k_{in}}{l}B-\varepsilon d_{b}B_{in}, \end{array}$$ (26)

$$\begin{array}{ccc} \frac{dP}{d\tau } & = & \varepsilon \nu d_{b}B_{in}-\varepsilon d_{p}P, \end{array}$$ (27)

whilst initial conditions remain the same as those given in Eq (21).

Using this reduced system we look to find the inner solutions of the system. We propose asymptotic solutions of the form *P*(*τ*; *ε*) = ∑_*n* = 0_ *ε*^*n*^ *P*^*n*^, *m*(*τ*; *ε*) = ∑_*n* = 0_ *ε*^*n*^ *m*^*n*^, *V*(*τ*; *ε*) = ∑_*n* = 0_ *ε*^*n*^ *V*^*n*^ and *F*(*τ*; *ε*) = ∑_*n* = 0_ *ε*^*n*^ *F*^*n*^ and equate coefficients of powers of epsilon to look for the inner solutions for free polymersomes and free receptors at early time points. For coefficients of *ε*^0^, the solutions for *m* and *P* are *m* = 1 and *P* = 0, as given by the initial conditions. These solutions allow us to solve the leading order solution for free receptors, *F*, and free polymersomes, *V*, with the equations for bound polymersomes (*B*) and internalised polymersomes (*B*_*in*_) decoupling from the rest of the system. For coefficients of order zero the equation for free polymersomes, (23), yields

$$\begin{array}{c} \frac{dV^{0}}{d\tau }=0. \end{array}$$ (28)

Solving this with the initial condition *V*(0) = *V*_0_, the leading order solution is then given simply by

$$\begin{array}{c} V^{0}\left(\tau \right)=V_{0}. \end{array}$$ (29)

Using this solution, we now look for the leading order solution for free receptors. Eq (24) becomes

$$\begin{array}{c} \frac{dF^{0}\left(\tau \right)}{d\tau }=-\left(\eta lk_{a}V^{0}\left(\tau \right)-d_{f}\right)F^{0}\left(\tau \right)+Rm, \end{array}$$ (30)

which is easily solved using the integration factor method with initial condition *F*^0^(0) = *F*_0_ to give

$$\begin{array}{c} F^{0}\left(\tau \right)=\frac{R}{\beta }+C_{1}e^{-\beta \tau }, \end{array}$$ (31)

where

$$\begin{array}{c} \beta =k_{a}\eta lV_{0}+d_{f}, \end{array}$$ (32)

and *C*_1_ is a constant of integration,

$$\begin{array}{c} C_{1}=F_{0}-\frac{R}{\beta }. \end{array}$$ (33)

During this time period free polymersomes become bound to cells (see Fig 3). Thus the number of free polymersomes *V*_0_(*t*) should not be equal to a constant. Equating coefficients of powers of *ε* for the free polymersome equation gives

$$\begin{array}{c} \frac{dV^{1}\left(\tau \right)}{d\tau }=-k_{a}V_{0}(\frac{R}{\beta }+C_{1}e^{-\beta \tau }), \end{array}$$ (34)

which can be easily solved to give

$$\begin{array}{c} V^{1}\left(\tau \right)=-k_{a}V_{0}(\frac{R}{\beta }+C_{1}e^{-\beta \tau })+C_{2}, \end{array}$$ (35)

where *C*_2_ is a constant of integration, given by

$$\begin{array}{c} C_{2}=-\frac{k_{a}V_{0}C_{1}}{\beta }. \end{array}$$ (36)

Next we find solutions for bound and internalised polymersomes. Recalling the solutions for free polymersomes and receptors, (29) and (31), and the equation for bound polymersomes (25) we have

$$\begin{array}{c} \frac{dB^{0}}{d\tau }=k_{a}lV_{0}(\frac{R}{\beta }+C_{1}e^{-\beta \tau })-k_{in}B\left(\tau \right). \end{array}$$ (37)

Again we can solve for *B*(*t*) using the integration factor method to give,

$$\begin{array}{c} B^{0}\left(\tau \right)=k_{a}lV_{0}(\frac{R}{k_{in}\beta }+\frac{C_{1}}{k_{in}-\beta }e^{-\tau \beta })+C_{3}e^{-k_{in}\tau } \end{array}$$ (38)

where *C*_3_ is a constant of integration,

$$\begin{array}{c} C_{3}=-k_{a}lV_{0}(\frac{R}{k_{in}\beta }+\frac{C_{1}}{k_{in}-\beta }). \end{array}$$ (39)

We now use the solution for bound polymersomes, (38), to solve for internalised polymersomes.

By combining the equation for *B*_*in*_(*τ*) (26) and by using (31), leads to

$$\begin{array}{c} \frac{dB_{in}}{d\tau }=\gamma_{1}+\gamma_{2}e^{-\beta t}+\gamma_{3}e^{-k_{in}t}+C_{3}e^{-k_{in}t}-d_{b}B_{in} \end{array}$$ (40)

where the following parameter groupings have been used,

$$\begin{array}{c} \gamma_{1}=\frac{k_{a}V_{0}R}{\beta }, \end{array}$$ (41)

$$\begin{array}{c} \gamma_{2}=\frac{k_{in}k_{a}V_{0}C_{1}}{k_{in}-\beta }, \end{array}$$ (42)

$$\begin{array}{c} \gamma_{3}=\frac{k_{in}C_{3}}{l}. \end{array}$$ (43)

Again this can be solved using the integration factor method, which yields

$$\begin{array}{c} B_{in}=\frac{\gamma_{1}}{d_{b}}+\frac{\gamma_{2}e^{-\beta \tau }}{d_{b}-\beta }+\frac{\gamma e^{-k_{in}\tau }}{d_{b}-k_{in}}+C_{4}e^{-d_{b}\tau }, \end{array}$$ (44)

where *C*_4_ is a factor of integration,

$$\begin{array}{c} C_{4}=-\frac{\gamma_{1}}{d_{b}}-\frac{\gamma_{2}}{d_{b}-\beta }-\frac{\gamma_{3}}{d_{b}-k_{in}}, \end{array}$$ (45)

**Fig 4:**
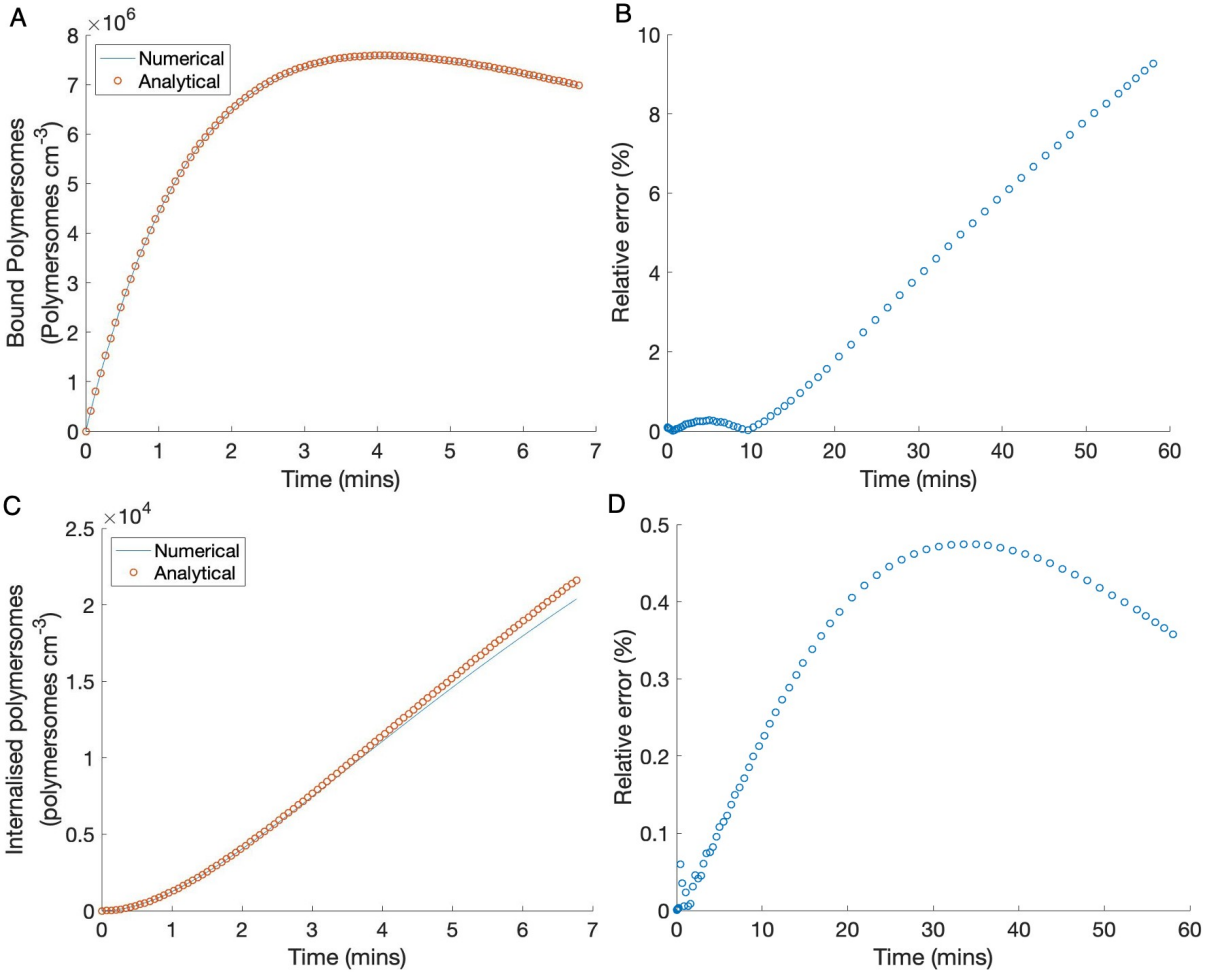
Numerical and analytical solutions for (A) bound and (C) internalised polymersomes for up to seven minutes and (B,D) the relative error in the numerical and analytical solutions for bound and internalised polymersomes respectively for up to one hour. The numerical solution is the original model but with some simplifying assumptions, given by Eqs 25 and 26, and the analytical solutions are derived from these via a perturbation analysis. The analytical solutions are given by Eqs 38 and 44. The nondimensional system and parameters were used for the simulations and the results were converted to dimensional values. The parameters used are given in Table 1.

The numerical and analytical results for bound and internalised nanoparticles are shown in Fig 4(A) and 4(C) along with the relative error between the solutions (B,D). We find a good match between the dimensionless numerical and analytical solutions at early time points which is demonstrated by the relative error between the solutions. The numerical and analytical solutions are shown for up to seven minutes in order to capture the key behaviour at early time points, that is that the maximum number of bound polymersomes occurs at a turning point, but the relative error is shown for a longer time period to demonstrate the validity of the results.

The relative error for bound and internalised polymersomes is shown in Fig 4 for the time period 0 ≤ *t* ≤ 60 minutes. For the bound polymersomes we see that for the first twenty minutes of the simulation the relative error is a few percent, after which the error grows with time. For the internalised polymersomes the relative error remains below 0.5%. Therefore, we take the analytical solutions to be acceptable for early time periods, less than ten minutes, in the model.

### 2.5 Maximising polymersome uptake

In Fig 4 we observe that the maximum number of bound polymersomes occurs at a turning point so now we look for an approximated analytical value of this maximum.

Differentiating (38) and solving at zero, the turning point occurs at *t* = t*, where

$$\begin{array}{c} t=t^{*}=\frac{1}{\beta -k_{in}}\text{ln}(\frac{\beta k_{a}lV_{0}C_{1}}{k_{in}C_{3}\left(\beta -k_{in}\right)}). \end{array}$$ (46)

therefore the maximum of number bound polymersomes, *B*^*max*^, is then given by

$$\begin{array}{c} B^{max}=B\left(t^{*}\right)=k_{a}lV_{0}(\frac{R}{k_{in}\beta }+\frac{C_{1}}{k_{in}-\beta }{\left(\frac{k_{in}C_{3}\left(\beta -k_{in}\right)}{\beta k_{a}lV_{0}l}\right)}^{\frac{\beta }{\beta -k_{in}}})+C_{3}{\left(\frac{k_{in}C_{3}\left(\beta -k_{in}\right)}{\beta k_{a}lV_{0}C_{1}}\right)}^{\frac{k_{in}}{\beta -k_{in}}}. \end{array}$$ (47)

Similarly, we can find the maximum of internalised polymersomes. We see from Fig 4 that at early time points the concentration of internalised polymersomes increases with time. Therefore we find the solution for internalised polymersomes as *τ* → ∞

$$\begin{array}{c} B_{in}^{max}=\frac{\gamma_{1}}{d_{b}} \end{array}$$ (48)

We use this result to investigate the importance of different parameters on polymersome internalisation which subsequently affects the delivered chemotherapy drug concentration.

### 2.6 Parameter dependency of maximum bound and internalised polymersomes

**Fig 5:**
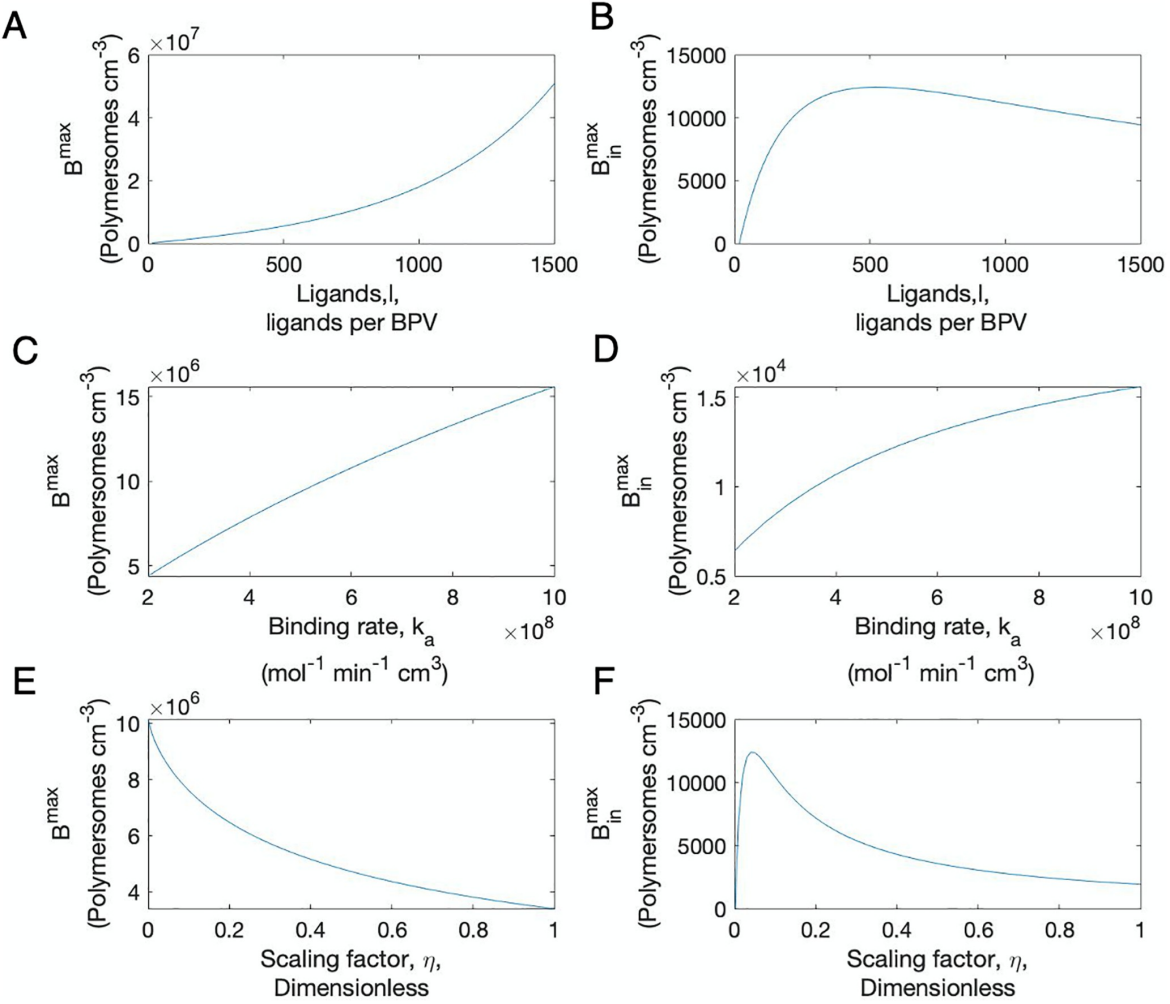
Sensitivity of the analytical solution of the maximum bound polymersomes and maximum internalised polymersomes, given by *B*^*max*^ (first column) and $B_{in}^{max}$ (second column), to variations in ligand number, *l* (first row), binding rate, *k*_*a*_, (second row) and the dimensionless scaling parameter, *η*, (third row). The nondimensional system and parameters were used for the simulations and the results were converted to dimensional values. The parameters used are given in Table 1.

Fig 3 reveals rapid system dynamics at early time points. To understand this behaviour in more detail we performed a singular perturbation analysis, the predictions for which are shown in Fig 4. From the analytical results we derived expressions for the maximum bound and internalised polymersomes. Fig 5 shows the behaviour of these quantities when we vary key parameters. In Fig 5, we observed that *B*^*max*^ is an increasing function of *l*, the fixed number of ligands per polymersome. The internalised polymersomes initially increases as *l* increases to reach a peak at approximately 500 ligands per polymersome before decreasing. As *l* continues to increase the internalisation rate by tumour cells slows, indicating that there is an optimal number of ligands to maximise polymersome internalisation. This is in agreement with the work of Ghaghada *et al*. [21] who also found that a specific number of ligands maximises polymersome uptake. *B*^*max*^ and $B_{in}^{max}$ are both increasing saturating functions of *k*_*a*_, meaning with increasing *k*_*a*_ more polymersomes become internalised. On the other hand *B*^*max*^ is a decreasing saturating function of the scaling factor, *η*. The maximum number of internalised polymersomes increases rapidly with *η* before decreasing, indicating an optimal value.

Next we apply our model to a spheroid system in order to incorporate spatial effects of polymersome delivery and to explore the impact of various internalisation factors on tumour growth, as well as tissue properties such as permeability.

## 3 Spheroid model

Tumour spheroids are an alternative *in vitro* preclinical screening tool for cancer drug development in addition to 2D monolayer experiments. Cells are seeded in a specially designed well; the cells cannot bind to the surface of the well so bind to each other and as they multiply they grow in a spheroid shaped mass. Initially the seeded cells have plenty of oxygen and nutrients to proliferate. However, oxygen and nutrient supply is diffusion-limited, so as the cell mass increases, some cells in the centre of the spheroid become located beyond the supply of nutrients and oxygen required for respiration. This leads to a formation of a quiescent layer of cells that receive enough nutrients and oxygen to survive but not to proliferate and at the centre of the spheroid no such supply is received and so cells die, forming a necrotic core. The spheroid geometry also limits drug penetration. The growth of the spheroid therefore leads to gradients in oxygen, nutrients and drugs from the surface towards the core.

**Fig 6:**
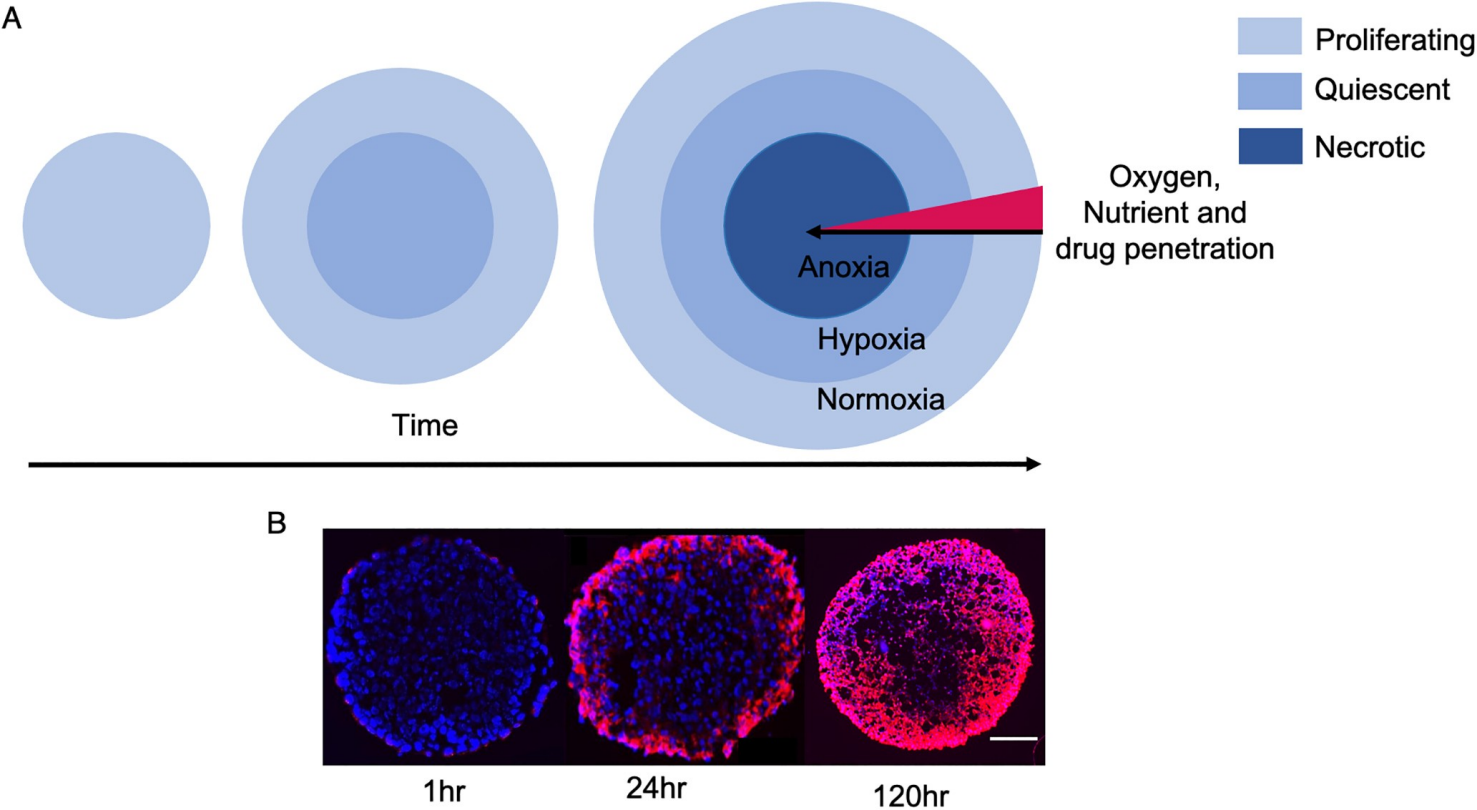
**(A)** Tumour spheroids grow from proliferating cells. As they grow over time quiescent and necrotic layers form due to gradients in oxygen and nutrients. **(B)** An example of nanoparticle penetration over time in tumour spheroids. The images shown are slices through a HNSCC tumour spheroid. Nanoparticles are rhodamine labelled PMPC-PDPA polymersomes. (left) 1 hour (middle) 24 hours, (right) 120 hours. Rhodamine fluorescence (red) and DAPI counterstained cell nuclei (blue). Scale bar = 100*μm*.

Fig 6 shows a schematic of tumour spheroid growth with oxygen, nutrient and drug gradients as well as some example experimental results of nanoparticle penetration into tumour spheroids over a period of 120hrs. It can been seen that it takes almost 24 hours for drugs to penetrate the outer layer of the spheroid. At 120 hours the outer and middle layer of the spheroid are permeated with nanoparticles but the centre is mostly void of them.

Here we extend our monolayer model of nanoparticle delivery to cells to describe uptake in a tumour spheroid, thus incorporating spatial effects. First we present the model, then explain the basis of the functional dependencies in the constitutive relationships. We then present the initial and boundary conditions of the model as well as any additional parameter values (see Table 2) followed by nondimensionalisation. This allows us to study the impact of various internalisation functions on spheroid growth over various time periods and various tissue properties, such as permeability.

**Table 2:** Parameter values used in the spheroid model not already defined in Table 1.

Parameter	Dimensional Value	Dimensionless Value	Reference
Random cell motility coefficient, *D*_*m*_	-	10	[29]
Random cellular material motility coefficient, *D*_*w*_	-	500	[29]
Oxygen diffusion coefficient, *D*_*c*_	1 × 10^−6^cm^2^s^−1^	4 × 10^3^	[19]
polymersome diffusion coefficient, *D*_*v*_	-	500	estimate
Maximum oxygen uptake by cells, $p_{cm}^{max}$	-	0.05	[17]
Maximum oxygen uptake due to proliferation, $p_{cp}^{max}$		0.014	[17]
Maximum cell growth rate, $p_{m}^{max}$	6.944 × 10^−4^min^−1^	-	[17]
Maximum cell death rate, $d_{m}^{max}$	-	2	[29]
Oxygen concentration for half maximal cell proliferation, *c*_*p*_	-	0.6	[17]
Oxygen concentration for half maximal cell death, *c*_*c*_	-	0.6	[17]
Potentcy, *γ*		10^−3^	estimate
Total volume fraction of cells and cellular material, *N*_0_	-	1	-
Radius of a cell, *R*_*m*_	6*μ*m	-	[29]

### 3.1 Mathematical formulation

We develop the model to describe radially symmetric avascular tumour growth with infiltrating polymersomes. The space is multiphase, accounting for cells and the surrounding material such as water and cell debris, which is reflective of experimental conditions. The model incorporates the binding kinetics and release of polymersome carried chemotherapy drug and so we can predict its associated impact on tumour growth.

In this model we do not account for certain physiochemical aspects of the nanoparticles, such as the variability in size, and the electric or magnetic properties. Sorrell et al [15] accounted for the variable sizes of the polymersomes, which occurs as a result of the production process, through the use of a stochastic model. The coating of polymersomes is chosen specifically for biological environments such that they do not react with the environment in any adverse way and so investigating these properties is beyond the scope of this work.

To setup the model we use dimensional parameters. The spheroid is a spherically symmetric sphere of radius, *R*. We assume the spheroid is comprised of two continuous phases, tumour cells and cellular material, as is common in the literature [17, 22, 28]. These phases are described by the volume fraction per unit control volume of tumour cells, *m*(*r*, *t*) and cellular material, *w*(*r*, *t*), which are functions of the distance from the spheroid centre, *r*, and time, *t*. We also include transport of oxygen, *c*(*r*, *t*), via diffusion from the culture media surrounding the spheroid, as well as distribution and binding of polymersomes. In order to simplify the model we neglect the chemotherapy drug components and instead calculate cell death as a function of polymersome concentration.

We apply a no-void condition within the spheroid, representing conservation of mass, hence *m* + *w* = *N*_*o*_, where *N*_*o*_ is a constant, given that *m* and *w* are normalised to a control volume of tumour cells.

The system is augmented via transport equations for the distribution of polymersomes, giving

$$\begin{array}{cc} \text{Tumour:}\;\\ & \frac{\partial m}{\partial t}=-\frac{1}{r^{2}}\frac{\partial }{\partial r}(r^{2}J_{m})+p_{m}\left(w,c\right)m-d_{m}\left(c\right)m-g\left(B\right), \end{array}$$ (49)

$$\begin{array}{cc} \text{Material:}\;\\ & \frac{\partial w}{\partial t}=-\frac{1}{r^{2}}\frac{\partial }{\partial r}(r^{2}J_{w})-p_{m}\left(w,c\right)m+d_{m}\left(c\right)m+g\left(B_{i}\right), \end{array}$$ (50)

$$\begin{array}{cc} \text{Oxygen:}\;\\ & \frac{\partial c}{\partial t}=-\frac{1}{r^{2}}\frac{\partial }{\partial r}(r^{2}J_{c})-d_{c}\left(m,w\right), \end{array}$$ (51)

$$\begin{array}{cc} \text{Free}\;\text{polymersomes:}\;\\ & \frac{\partial V}{\partial t}=-\frac{1}{r^{2}}\frac{\partial }{\partial r}(r^{2}J_{v})-k_{a}VF+\frac{k_{d}}{l}B_{1}, \end{array}$$ (52)

$$\begin{array}{cc} B_{1}\\ & \frac{\partial B_{1}}{\partial t}=-\frac{1}{r^{2}}\frac{\partial }{\partial r}(r^{2}\frac{B_{1}}{m}J_{m})+k_{a}lVF-\left(k_{d}+k_{in}\left(1\right)\right)B_{1}\\ & \;-k_{a}\rho_{f}(\rho_{l}lN_{a}-1)FB_{1}+2k_{d}B_{2}, \end{array}$$ (53)

$$\begin{array}{cc} B_{i}’s:\\ & \frac{\partial B_{i}}{\partial t}=-\frac{1}{r^{2}}\frac{\partial }{\partial r}(r^{2}\frac{B_{i}}{m}J_{m})+k_{a}\rho_{f}(\rho_{l}lN_{a}-\left(i-1\right))FB_{i-1}\\ & \;-\left(ik_{d}+k_{in}\left(i\right)\right)B_{i}-k_{a}\rho_{f}(\rho_{l}lN_{a}-\left(i-1\right))FB_{i}\\ & \;+\left(i+1\right)k_{d}B_{i+1},\;\text{for}\;2\leq i\leq n-1, \end{array}$$ (54)

$$\begin{array}{cc} B_{n}\\ & \frac{\partial B_{n}}{\partial t}=-\frac{1}{r^{2}}\frac{\partial }{\partial r}(r^{2}\frac{B_{n}}{m}J_{m})+k_{a}\rho_{f}(\rho_{l}lN_{a}-\left(n-1\right))FB_{n-1}\\ & \;-\left(nk_{d}+k_{in}\left(n\right)\right)B_{n}, \end{array}$$ (55)

where *J*_*m*_, *J*_*w*_, *J*_*c*_ and *J*_*v*_ are the fluxes of tumour cells, cellular material, oxygen and free polymersomes, respectively. The fluxes are given by

$$\begin{array}{c} J_{m}=-D_{m}\frac{\partial m}{\partial r}+vm,\;J_{w}=-D_{w}\frac{\partial w}{\partial r}+vw,\\ J_{c}=-D_{c}\frac{\partial c}{\partial r}+vc,\;J_{v}=-D_{v}\frac{\partial V}{\partial r}+vV, \end{array}$$ (56)

where **v** is the common advection velocity (the velocity of all constituents in the model due to bulk motion) and *D*_*m*_, *D*_*w*_, *D*_*c*_, *D*_*v*_ are the diffusivities of the tumour cells, cellular material, oxygen and free polymersomes respectively. We assume that once the polymersomes have bound to the cell then they move with the cell. Using the no-void condition and by summing Eqs (49) and (50) we can deduce the following advection velocity,

$$\begin{array}{c} v=\frac{1}{N_{0}}((D_{m}-D_{w})\frac{\partial m}{\partial r}). \end{array}$$ (57)

Next, we discuss the functional forms of the terms in the model. The framework we are using here has been adapted from similar work which models the use of therapeutic macrophages by Webb et al [17]. The function *p*_*m*_(*w*, *c*) denotes tumour proliferation, which we take to be an increasing saturating function of both the cellular material and oxygen concentration,

$$\begin{array}{c} p_{m}\left(w,c\right)=p_{m}^{max}S\left(c,c_{p}\right)\text{min}(\frac{w}{w_{0}},1), \end{array}$$ (58)

and the death rate due to apoptosis, *d*_*m*_(*c*), is a decreasing function of the oxygen concentration,

$$\begin{array}{c} d_{m}\left(c\right)=d_{m}^{max}\left(1-S\left(c,c_{c}\right)\right). \end{array}$$ (59)

We capture oxygen metabolism through the function *d*_*c*_(*m*, *w*, *c*) which increases with tumour cell density,

$$\begin{array}{c} d_{c}\left(m,w,c\right)=p_{cm}^{max}S\left(c,c_{c}\right)m+p_{cp}^{max}p_{m}\left(w,c\right)m. \end{array}$$ (60)

where

$$\begin{array}{c} S\left(c,c_{p}\right)=\frac{c^{\alpha }\left(c_{p}^{\alpha }+c_{\infty }^{\alpha }\right)}{c_{\infty }^{\alpha }\left(c_{p}^{\alpha }+c^{\alpha }\right)}, \end{array}$$ (61)

*c*_*p*_ is the oxygen threshold for proliferation, *c*_*c*_ is the oxygen concentration for half maximal cell death, $p_{cm}^{max}$ is the maximum uptake rate of oxygen by cells, $p_{cp}^{max}$ is the maximum uptake rate of oxygen due to proliferation and $p_{m}^{max}$ is the maximum cell growth rate under nutrient rich conditions. *S*(*c*, *c*_*p*_) is a scaled Hill function with maximal value *S* = 1 when *c* = *c*_∞_ (which we assume is the maximum value of oxygen at the tumour boundary), with the steepness of the curve dependent on the value of the exponent *α* > 0.

For simplicity the death of cells is a calculated as function of bound particles, reducing the number of calculations needed in the model. We assume that the death rate of tumour cells, *g*(*B*), is proportional to the total number of bound polymersomes,

$$\begin{array}{c} g\left(B\right)=\gamma \sum_{i=1}^{n}k_{in}B_{i}, \end{array}$$ (62)

where *γ* is the potency of the polymersomes. To further simplify the framework, we assume a fixed number of receptors per cell, *f*_0_. So that, at any time, the free receptors per cell is given by this fixed number minus those involved in polymersome binding, namely

$$\begin{array}{c} F=\underset{\begin{array}{c} \text{total}\;\text{mol}\;\text{receptors}\\ \text{per}\;{\text{cm}}^{3} \end{array}}{\overset{︷}{f_{0}m}}-\underset{\begin{array}{c} \text{mol}\;\text{ligand-receptor}\\ \text{complexes}\;\text{per}\;{\text{cm}}^{3} \end{array}}{\overset{︷}{\sum_{i=1}^{n}iB_{i}}} \end{array}$$ (63)

Using this model set up, we can solve Eqs (53)–(55) independently to give numerical equations for polymersomes with different bond numbers, *i*.*e* for each *B*_*i*_, (where *i* = 1, …, *n*), in terms of the remaining model variables. It is useful to solve for each *B*_*i*_ because this allows us to perform investigations into how the number of bonds between polymersomes and cells effects possible outcomes of spheroid growth. The values of the parameters that are introduced in this section are given in Table 2, the rest of the parameters which were also used in the previous section remain the same and are given in Table 1.

### 3.2 Initial and boundary conditions

Next we set the initial and boundary conditions for the model. Initially, the tumour is allowed to grow without the presence of therapeutic polymersomes, as the spheroid would in experiments, therefore at *t* = 0

$$\begin{array}{cc} & m\left(r,0\right)=M_{0},\;w\left(r,0\right)=N_{0}-M_{0},\;c\left(r,0\right)=c_{0}, \end{array}$$ (64)

$$\begin{array}{cc} & V\left(r,0\right)=0,\;B\left(r,0\right)=0,\;V_{\infty }=0\;\text{and}\;R\left(0\right)=R_{0}, \end{array}$$ (65)

where *V*_∞_ is the polymersome surface concentration, *M*_0_ is the tumour cell density, *c*_0_ is the oxygen concentration, *B*(*r*, 0) is the bound polymersome concentration and *V*(*r*, 0) is the polymersome concentration at distance r from the spheroid centre and *R*_0_ is the initial radius of the spheroid. At some time, t*, we introduce the polymersomes on the surface of the tumour spheroid by setting the surface concentration of polymersomes, to some non-zero value (previously *V*_∞_ = 0 when 0 < *t* < t*).

Assuming the tumour is symmetric about the origin, we have

$$\begin{array}{c} \frac{\partial m}{\partial r}=\frac{\partial w}{\partial r}=\frac{\partial c}{\partial r}=\frac{\partial V}{\partial r}=\frac{\partial B}{\partial r}=0\;\text{on}\;r=0. \end{array}$$ (66)

On the tumour boundary *r* = *R*(*t*), we fix the nutrient concentration, *c* = *c*_∞_. Both cellular material and polymersomes can move across the tumour boundary with their flux across the boundary proportional to (*w*_∞_ − *w*(*R*, *t*)) and (*V*_∞_ − *V*(*R*, *t*)), respectively, where *w*_∞_ is the external concentration of cellular material. The tumour boundary moves with the velocity **v**_*m*_ and cellular material moves across the boundary at velocity **v**_*w*_, hence the flux boundary conditions for cellular material and unbound polymersomes on *r* = *R*(*t*) are given by,

$$\begin{array}{ccc} -w(v_{w}-v_{m}) & = & h_{w}\left(w_{\infty }-w\right)\\ & \Rightarrow & D_{w}\frac{\partial m}{\partial r}-D_{m}\frac{\left(N_{o}-m\right)}{m}\frac{\partial m}{\partial r}=h_{w}\left(w_{\infty }-N_{o}+m\right)|{}_{r=R(t)}, \end{array}$$ (67)

$$\begin{array}{ccc} -V(v_{v}-v_{m}) & = & h_{v}\left(V_{\infty }-V\right)\\ & \Rightarrow & D_{v}\frac{\partial V}{\partial r}-D_{m}\frac{V}{m}\frac{\partial m}{\partial r}=h_{v}\left(V_{\infty }-V\right)|{}_{r=R(t)}, \end{array}$$ (68)

where *h*_*w*_ and *h*_*v*_ are the positive permeabilities of the cellular material and free polymersomes, respectively, across the tumour boundary. The velocities of tumour cells, **v**_*m*_, cellular material, **v**_*c*_, and free polymersomes, **v**_*v*_, are given by

$$\begin{array}{c} v_{m}=v-\frac{D_{m}}{m}\frac{\partial m}{\partial r},\;v_{w}=v-\frac{D_{w}}{w}\frac{\partial w}{\partial r},\;v_{v}=v-\frac{D_{v}}{V}\frac{\partial V}{\partial r}. \end{array}$$

The boundary conditions for bound polymersomes, *B*_*i*_’s, on *r* = 0 and *r* = *R*(*t*) can be found assuming the bound polymersomes are in quasi steady state (discussed in section 3.4).

The spheroid boundary moves with the tumour velocity **v**_*m*_,

$$\begin{array}{c} \frac{dR}{dt}=v_{m}{|}_{r=R(t)}=(v-\frac{D_{m}}{m}\frac{\partial m}{\partial r}){|}_{r=R(t)}. \end{array}$$ (69)

Using (57) we can re-write (69) as

$$\begin{array}{c} \frac{dR}{dt}=\frac{1}{N_{o}}h_{w}\left(w_{\infty }+m-N_{0}\right). \end{array}$$ (70)

Similarly to the well-mixed system we nondimensionalise due to the large variation in the magnitude of parameter values and to allow us to identify dominant terms in the model.

### 3.3 Nondimensionlisation

In this section, the system of Eqs (49)–(55) is nondimensionalised alongside the counterpart initial and boundary conditions to evaluate the dominant balance of different mechanisms. We use the following re-scalings to nondimensionalise the system, where the hats denote the nondimensional variables,

$$\begin{array}{c} m=\hat{m}N_{o},\;w=\hat{w}N_{o},\;c=\hat{c}c_{\infty },\;V=\frac{\hat{V}}{v_{m}},\;B=\frac{\hat{B}}{v_{m}},\;F=\frac{\hat{F}}{v_{m}}, \end{array}$$

$$\begin{array}{c} r=\hat{r}R_{m},\;v=\hat{v}R_{m}p_{m}^{max},\;\hat{l}=lN_{a},\;\hat{t}=p_{m}^{max}t, \end{array}$$

where *v*_*m*_ is the volume of a tumour cell, *R*_*m*_ is the radius of an individual tumour cell and *c*_∞_ is the room temperature atmospheric oxygen pressure. On dropping the hats we obtain

$$\begin{array}{ccc} \frac{\partial m}{\partial t} & = & -\frac{1}{r^{2}}\frac{\partial }{\partial r}(r^{2}{\tilde{J}}_{m})+{\tilde{p}}_{m}\left(w,c\right)m-{\tilde{d}}_{m}\left(c\right)m-\tilde{g}\left(B\right), \end{array}$$ (71)

$$\begin{array}{ccc} \frac{\partial c}{\partial t} & = & -\frac{1}{r^{2}}\frac{\partial }{\partial r}(r^{2}{\tilde{J}}_{c})-{\tilde{d}}_{c}\left(m,w\right), \end{array}$$ (72)

$$\begin{array}{ccc} \frac{\partial V}{\partial t} & = & -\frac{1}{r^{2}}\frac{\partial }{\partial r}(r^{2}{\tilde{J}}_{v})-{\tilde{k}}_{a}V\left({\tilde{f}}_{0}-b\right)+\frac{{\tilde{k}}_{d}}{l}B_{1}, \end{array}$$ (73)

$$\begin{array}{ccc} \frac{\partial B_{1}}{\partial t} & = & -\frac{1}{r^{2}}\frac{\partial }{\partial r}(r^{2}\frac{B_{1}}{m}{\tilde{J}}_{m})+{\tilde{k}}_{a}lVF-\left({\tilde{k}}_{d}+{\tilde{k}}_{in}\left(1\right)\right)B_{1}\\ & & -{\tilde{k}}_{a}^{d}\rho_{f}(\rho_{l}lN_{a}-1)FB_{1}+2{\tilde{k}}_{d}B_{2}, \end{array}$$ (74)

$$\begin{array}{ccc} \frac{\partial B_{i}}{\partial t} & = & -\frac{1}{r^{2}}\frac{\partial }{\partial r}(r^{2}\frac{B_{i}}{m}{\tilde{J}}_{m})+{\tilde{k}}_{a}\rho_{f}(\rho_{l}lN_{a}-\left(i-1\right))FB_{i-1}-\\ & & \left(i{\tilde{k}}_{d}+{\tilde{k}}_{in}\left(i\right)\right)B_{i}-{\tilde{k}}_{a}\rho_{f}(\rho_{l}lN_{a}-\left(i-1\right))FB_{i}+\\ & & \left(i+1\right){\tilde{k}}_{d}B_{i+1},\;\text{for}\;2\leq i\leq n-1, \end{array}$$ (75)

$$\begin{array}{ccc} \frac{\partial B_{n}}{\partial t} & = & -\frac{1}{r^{2}}\frac{\partial }{\partial r}(r^{2}\frac{B_{n}}{m}{\tilde{J}}_{m})+{\tilde{k}}_{a}\rho_{f}(\rho_{l}lN_{a}-\left(n-1\right))FB_{n-1}-\\ & & \left(n{\tilde{k}}_{d}+{\tilde{k}}_{in}\left(n\right)\right)B_{n}, \end{array}$$ (76)

where

$$\begin{array}{c} {\tilde{p}}_{m}=S\left(\hat{c},{\tilde{c}}_{p}\right)\min(\frac{w}{{\tilde{w}}_{0}},1),\;{\tilde{d}}_{m}={\tilde{d}}_{m}^{max}\left\{1-S\left(\hat{c},{\tilde{c}}_{c}\right)\right\},\;{\tilde{d}}_{c}\left(m,w\right)=\frac{d_{c}\left(m,w\right)}{D_{c}c_{\infty }}, \end{array}$$

$$\begin{array}{c} \tilde{g}\left(B\right)=\tilde{\gamma }\sum_{i=1}^{n}{\tilde{k}}_{in}B_{i},\;S\left(\hat{c},{\tilde{c}}_{p}\right)=\frac{c^{\alpha }\left({\tilde{c}}_{p}^{\alpha }+1\right)}{\left({\tilde{c}}_{p}^{\alpha }+c^{\alpha }\right)} \end{array}$$

and

$$\begin{array}{c} {\tilde{J}}_{m}=-{\tilde{D}}_{m}\frac{\partial m}{\partial r}+vm, \end{array}$$

$$\begin{array}{c} {\tilde{J}}_{c}=-\frac{\partial c}{\partial r},\;{\tilde{J}}_{v}=-{\tilde{D}}_{v}\frac{\partial V}{\partial r}+vV. \end{array}$$

The non-dimensional advection velocity then is as follows

$$\begin{array}{c} v=({\tilde{D}}_{m}-{\tilde{D}}_{w})\frac{\partial m}{\partial r}. \end{array}$$ (77)

The parameter re-scalings read as,

$$\begin{array}{llll} {\tilde{D}}_{m}=\frac{D_{m}}{R_{m}^{2}p_{m}^{max}}, & {\tilde{D}}_{w}=\frac{D_{w}}{R_{m}^{2}p_{m}^{max}}, & {\tilde{D}}_{c}=\frac{D_{c}}{R_{m}^{2}p_{m}^{max}}, & {\tilde{D}}_{v}=\frac{D_{v}}{R_{m}^{2}p_{m}^{max}},\\ {\tilde{k}}_{in}(i)=\frac{k_{in}(i)}{p_{m}^{max}}, & {\tilde{k}}_{d}=\frac{k_{d}}{p_{m}^{max}}, & {\tilde{k}}_{a}=\frac{k_{a}N_{o}}{p_{m}^{max}v_{m}N_{a}}, & {\tilde{f}}_{0}=f_{0}N_{a},\\ \tilde{\gamma }=\frac{\gamma }{N_{a}}, & {\tilde{c}}_{p}=\frac{c_{p}}{c_{\infty }}, & {\tilde{c}}_{c}=\frac{c_{c}}{c_{\infty }}, & {\tilde{w}}_{0}=\frac{w_{0}}{N_{o}},\\ {\tilde{d}}_{m}^{max}=\frac{d_{m}^{max}}{p_{m}^{max}}. & & & \end{array}$$

For convenience in the remaining analysis we drop the tildes.

### 3.4 Numerical solutions

Due to the fast timescale of oxygen diffusion (*D*_*w*_ = 1 × 10^−6^cm^2^s^−1^) compared to the timescale of tumour growth (0.05cm/day) [19] we make the assumption that the oxygen concentration is at quasi -steady state, as is common in the literature [19, 30]. The binding kinetics of the receptors and ligands are fast compared to that of tumour cell growth, the binding rate, *k*_*a*_, is on the order of 10^8^mol^−1^min^−1^cm^3^ whereas cell division is on the order of 10^−4^min^−1^. This allows us to make the assumption that bound polymersomes are in quasi steady state compared to cell growth. We also note that the flux of cells is much lower than that of free nanoparticles and oxygen, so comparatively the cells are stationary on the tissue scale of cell movement, growth and decay. Thus the equations describing ligand-receptor complexes, Eqs (74)–(76), become

$$\begin{array}{ccc} 0 & = & k_{a}lVF-\left(k_{d}+k_{in}\left(1\right)\right)B_{1}-k_{a}\rho_{f}(\rho_{l}lN_{a}-1)FB_{1}+2k_{d}B_{2}, \end{array}$$ (78)

$$\begin{array}{ccc} 0 & = & k_{a}\rho_{f}(\rho_{l}lN_{a}-\left(i-1\right))FB_{i-1}-\left(ik_{d}+k_{in}\left(i\right)\right)B_{i}-k_{a}\rho_{f}(\rho_{l}lN_{a}-\left(i-1\right))FB_{i},\\ & & +\left(i+1\right)k_{d}B_{i+1},\;\;\;\text{for}\;i=2,...,n-1, \end{array}$$ (79)

$$\begin{array}{ccc} 0 & = & k_{a}\rho_{f}(\rho_{l}lN_{a}-\left(n-1\right))FB_{n-1}-\left(nk_{d}+k_{in}\left(n\right)\right)B_{n}, \end{array}$$ (80)

and the equation for oxygen, Eq 72, becomes

$$\begin{array}{c} 0=-\frac{1}{r^{2}}\frac{\partial }{\partial r}(r^{2}J_{c})-d_{c}\left(m,w\right). \end{array}$$ (81)

We can now solve analytically for each B_*i*_. First, we take *n* = 2, where the maximum number of bonds per polymersome is 2. Eqs (78)–(80) then give

$$\begin{array}{ccc} 0 & = & k_{a}lV\left(f_{0}m-B_{1}-2B_{2}\right)-\left(k_{d}+k_{in}\left(1\right)\right)B_{1}\\ & & -k_{a}\rho_{f}\left(\rho_{l}lN_{a}-1\right)(f_{0}m-B_{1}-2B_{2})B_{1}+2k_{d}B_{2}, \end{array}$$ (82)

$$\begin{array}{ccc} 0 & = & k_{a}\rho_{f}\left(\rho_{l}lN_{a}-1\right)\left(f_{0}m-B_{1}-2B_{2}\right)B_{1}\\ & & -\left(2k_{d}+k_{in}\left(2\right)\right)B_{2}. \end{array}$$ (83)

We can express the solution for *B*_1_ and *B*_2_ analytically in terms of *m* and *V*, namely,

$$\begin{array}{cc} B_{1}\left(r,t\right)\;= & \;\frac{1}{2\gamma_{1}\left(2\mu_{1}-\mu_{2}\right)}(-k_{a}lV\mu_{2}-\gamma_{1}k_{in}\left(2\right)f_{0}m-\mu_{1}\mu_{2}\\ & +(\gamma_{1}^{2}k_{in}{\left(2\right)}^{2}f_{0}^{2}m^{2}+2\gamma_{1}\mu_{2}f_{0}m(4k_{a}lV\mu_{1}\\ & -k_{a}lVk_{in}\left(2\right))+\mu_{2}^{2}{\left(k_{a}lV+\mu_{1}\right)}^{2}{)}^{\frac{1}{2}}), \end{array}$$ (84)

$$\begin{array}{c} B_{2}\left(r,t\right)\;=\;\frac{\gamma_{1}\left(B_{1}+f_{0}m\right)B_{1}}{2\gamma_{1}B_{1}+\mu_{2}}, \end{array}$$ (85)

where

$$\begin{array}{c} \gamma_{1}=k_{a}\rho_{f}\left(\rho_{l}l-1\right),\;\mu_{1}=k_{d}+k_{in}\left(1\right)\;\;\text{and}\;\;\mu_{2}=2k_{d}+k_{in}\left(2\right). \end{array}$$ (86)

Note that the algebraic system (78)–(80) with *n* = 3 is analytically intractable so we need to make an additional simplifying assumption by introducing an additional approximation to *F*. To do this we assume that there are many more free receptors than bound, so that $F\gg \sum_{i=1}^{n}iB_{i}$, where *i* = 1, …, *n*, and we can make the approximation

$$\begin{array}{c} F=f_{0}m, \end{array}$$ (87)

which allows us to calculate B_*i*_ for *n* > 2. We solve the system for up to *n* = 5 (equations not shown here for brevity).

With the solutions for *B*_*i*_(*r*, *t*) we solve the nondimensionalised system (Eqs 71–73) with the boundary conditions given by

$$\begin{array}{c} \frac{\partial m}{\partial r}=\frac{\partial c}{\partial r}=\frac{\partial V}{\partial r}\;\text{on}\;r=0. \end{array}$$ (88)

On the spheroid boundary, *r* = *R*(*t*), the oxygen concentration is *c* = 1 and we have

$$\begin{array}{c} -D_{n}\frac{\partial m}{\partial r}-D_{m}\frac{1-m}{m}\frac{\partial m}{\partial r}=h_{\infty }\left(n_{\infty }+m-1\right){|}_{r=R(t)}, \end{array}$$ (89)

$$\begin{array}{c} D_{v}\frac{\partial V}{\partial r}-D_{v}\frac{V}{m}\frac{\partial m}{\partial r}=h_{\infty }\left(V_{\infty }-V\right){|}_{r=R(t)}. \end{array}$$ (90)

The spheroid boundary (*r* = *R*(*t*)) now moves with the tumour velocity so that

$$\begin{array}{c} \frac{dR}{dt}=h_{n}\left(n_{\infty }+m-1\right). \end{array}$$ (91)

The system is solved numerically using NAG routine D03PHF which uses a finite difference approach to integrate over one spatial variable and the method of lines to reduce the PDEs to a system of ODEs. The resulting system is solved using a backward differentiation formula method.

### 3.5 Spheroid growth before, during and after polymersome application

**Fig 7:**
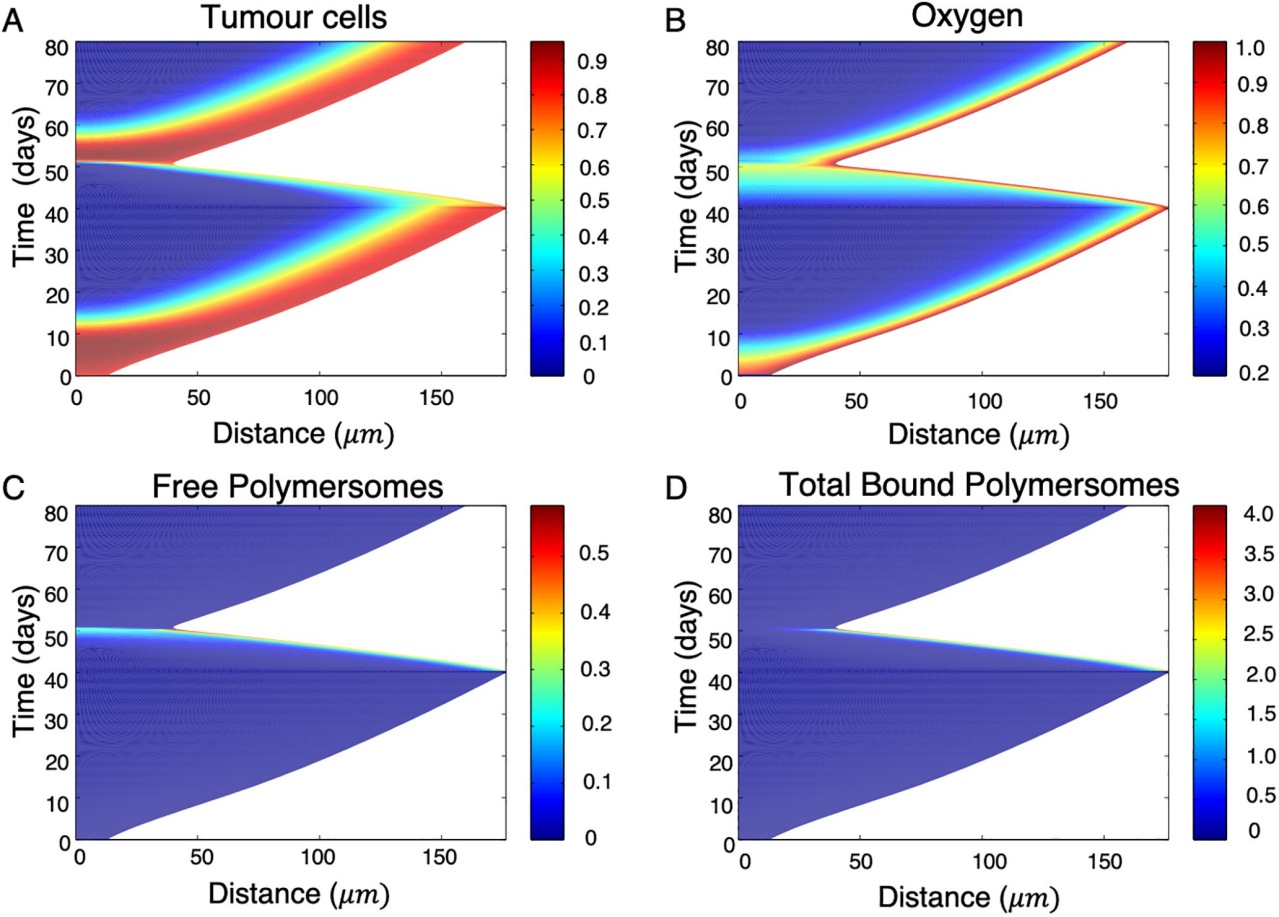
Evolution of a tumour spheroid and associated variables in time, including the tumour cell, oxygen, free and bound polymersome concentrations for *n* = 5. The cellular concentration is given as the volume fraction of live tumour cells. The rest of the concentrations are dimensionless. Initially growth occurs with no free polymersomes on the tumour surface up to a radius of 175*μm*, then we introduce polymersomes by setting *V*_∞_ = 10 when 40 ≤ *t* ≤ 50 days. The tumour growth rate decreases and the spheroid shrinks for the period of time when treatment is administered, but returns to linear growth (indicating travelling wave solutions) afterwards. The simulations were conducted using the dimensionless framework and the time and distance converted to dimensional form afterwards. The parameter values used are give in Tables 1 and 2.

With the system solved numerically we can investigate impact of applying polymersomes to tumour spheroid growth. Fig 7 shows the model predictions in space and time for tumour cell density, oxygen concentration and free and total bound polymersomes for a constant internalisation function and a maximum number of bonds, *n* = 5. The spheroid grows initially in the absence of oxidative stress. During this period we observe linear growth of the spheroid radius, with a high concentration of proliferating cells at the surface which rapidly decays to no proliferating cells towards the spheroid centre at *r* = 0, indicating a necrotic core due to lack of oxygen. At time t = 40 days polymersomes are applied to the surface of the spheroid, which are then removed at t = 50 days. During the period of polymersome application, we observe a noticeable decrease in spheroid radius. The polymersomes are confined to the boundary of the spheroid, where the chemotherapy drug is unloaded into proliferating cells, causing the spheroid to shrink. As would be expected, the total bound polymersomes follows a similar trend to free polymersomes. Once the polymersomes are removed the cells at the edge of the spheroid become re-oxygenated due to the smaller radius allowing for effective diffusion. This then provides a good environment for growth, hence the spheroid re-grows linearly for *t* > 50 days.

Next we investigate the model predictions using two different internalisation functions.

### 3.6 Effects of constant and bond dependent internalisation functions

It has been assumed so far in this paper, and in our previous work, that internalisation is constant. However, it is possible that internalisation is bond dependent so we also define a bond dependent internalisation function, in addition to the constant function. The bond dependent function is a simple step-wise Hill function

$$\begin{array}{c} k_{in}\left(i\right)=k_{in}(\varepsilon_{1}+H\left(i-j^{*}\right)), \end{array}$$ (92)

where *ε*_1_ is a small number. In this scenario polymersomes are not internalised until they reach the bond number specified by the activator, j*.

**Fig 8:**
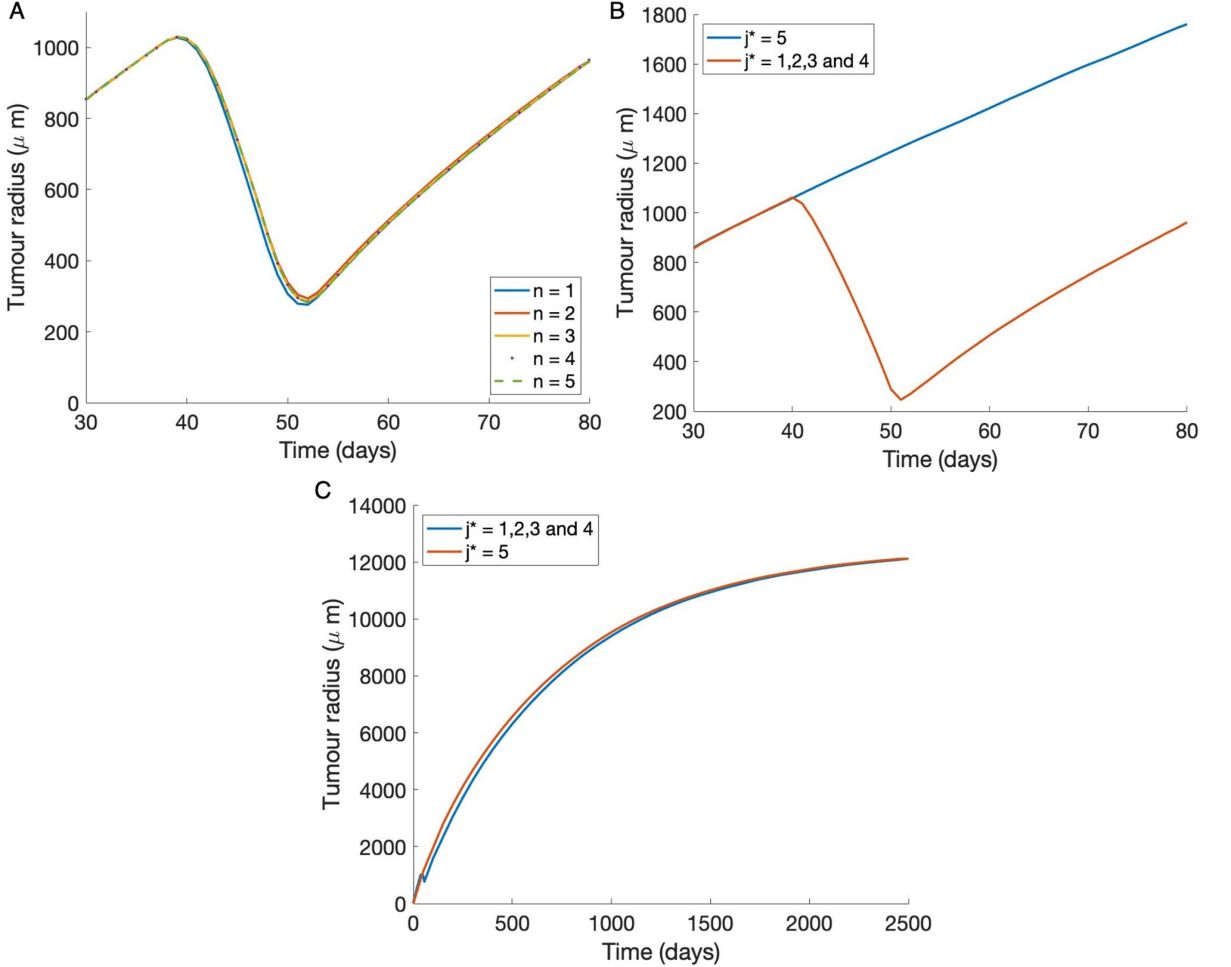
The impact of polymersomes on the spheroid dynamics using the numerical solutions for bonds. **(A)** Tumour radius dependency on the maximum number of bonds, *n*, for the constant internalisation function. The maximum bond number is varied from *n* = 1 ⋯ 5. **(B) (C)** The tumour radius with time for a bond dependent internalisation function for various values of the activator, j*. Polymersomes are applied to the tumour surface at 40 ≤ *t* ≤ 50 days with the parameters *V*_∞_ = 10, the rest of the parameter values used are give in Tables 1 and 2. **(B)** Polymersome application and growth over 80 days **(C)** Polymersome application and growth over a much longer time period of 2500 days.

In Fig 8A we show the model predictions for tumour radius using the constant internalisation function for *n* = 1 ⋯ 5. As before the tumour is allowed to grow until *t* = 40 days at which point polymersomes are applied until *t* = 50 days. We see very little variation in the predictions which implies internalisation happens quickly before multiple bonds can form. This is in agreement with our predictions in the well-mixed system (see Fig 3). In Fig 8B and 8C, we show the predictions for the case of the bond dependent function. Fig 8 indicates that when the activator, j* ≤ 4, there is a decrease in spheroid radius during the period of treatment comparable to that of constant internalisation, but if j* > 4 there is no reduction in spheroid growth during the treatment time. Therefore, with this set of parameters j* = 5 appears to be a threshold. That is, by forcing the polymersome to bind to at least 5 complexes before internalisation we slow down the internalisation of the polymersome sufficiently so that treatment is then not effective within this time frame. Fig 8 also shows longer term solutions, for 0 ≤ *t* ≤ 2500 days. We find that over long time periods the model predictions tend to the same solution.

### 3.7 Large time behaviour

In the absence of polymersomes the tumour spheroid appears to grow linearly (see Fig 7). In order to find exact solutions for this behaviour we look to the asymptotic solutions of the system as *t* → ∞ and hence we carry out a travelling wave analysis by assuming that the tumour grows with constant speed, *u* > 0.

The rate of change of the spheroid radius will then be

$$\begin{array}{c} \frac{dR}{dt}=u, \end{array}$$ (93)

so that *R* ∼ *ut* as *t* → ∞. We redefine our system with the travelling wave coordinate, *z* = *r* − *ut*, and rewrite the Eqs (49), (51) and (52) in terms of *z*:

$$\begin{array}{c} -um^{\prime }=\frac{2}{r}\left(D_{m}m^{\prime }-mv\right)+){\left(D_{m}m^{\prime }-mv\right)}^{\prime }+p_{m}\left(w,c\right)m-d_{m}\left(c\right)m-g\left(B\right), \end{array}$$ (94)

$$\begin{array}{c} c^{\prime \prime }=d_{c}\left(m,w\right), \end{array}$$ (95)

$$\begin{array}{c} -uV^{\prime }=\frac{2}{r}\left(D_{v}V^{\prime }-Vv\right)+{\left(D_{m}V^{\prime }-Vv\right)}^{\prime }-k_{a}VF+\frac{k_{d}}{l}B, \end{array}$$ (96)

where the prime denotes the derivative with respect to z. We use the quasi-steady state expressions for bound polymersomes as described in Eqs 84 and 85. The advection velocity can be written as

$$\begin{array}{c} v=\psi m^{\prime }\;\;\text{where}\;\;\psi =D_{m}-D_{w}. \end{array}$$ (97)

The system is represented by the following first order ODEs,

$$\begin{array}{ccc} m^{\prime } & = & W, \end{array}$$ (98)

$$\begin{array}{ccc} c^{\prime } & = & P, \end{array}$$ (99)

$$\begin{array}{ccc} V^{\prime } & = & Q, \end{array}$$ (100)

$$\begin{array}{ccc} W^{\prime } & = & \frac{1}{\psi m-d_{m}}(uW-\psi W^{2}+p_{m}\left(w,c\right)m-d_{m}\left(c\right)m-g\left(B_{i}\right)), \end{array}$$ (101)

$$\begin{array}{ccc} P^{\prime } & = & d_{c}\left(m,w\right), \end{array}$$ (102)

$$\begin{array}{ccc} Q^{\prime } & = & -\frac{1}{D_{v}}(\left(u-\psi W\right)Q-\psi WV-k_{a}VF+\frac{k_{d}}{l}B_{1}), \end{array}$$ (103)

for *i* = 1, …, *n*. Note that we have neglected the terms containing *r*^−1^ since they are $O(R^{-1})$ when *R* → ∞.

We solve this system of equations numerically using AUTO (a bifurcation and continuation software) [31]. To facilitate the solver we truncate the semi-finite domain $\hat{z}\in \left(-\infty ,0\right]$ to $\hat{z}\in \left[-R,100\right]$ where *R* > 0 and taken to be sufficiently large for AUTO to solve the boundary value problem.

We rescale *z*

$$\begin{array}{c} \hat{z}=\frac{z}{R}+100 \end{array}$$ (104)

so that $\hat{z}\in \left[0,100\right]$.

The wavespeed *u* can be written using 91

$$\begin{array}{c} u=h_{n}\left(n_{\infty }+m-1\right){|}_{\hat{(}z)=100}. \end{array}$$ (105)

The system is subject to the following boundary conditions on the truncated domain $\hat{z}\in \left[0,100\right]$: at $\hat{z}=0$

$$\begin{array}{c} m^{\prime }\left(0\right)=c^{\prime }\left(0\right)=V^{\prime }\left(0\right)=0; \end{array}$$ (106)

at $\hat{z}=100$,

$$\begin{array}{ccc} & & -D_{w}m^{\prime }-D_{m}\frac{\left(1-m\right)}{m}m^{\prime }=h_{w}\left(w_{\infty }+m-1\right)|{}_{\hat{z}=100}, \end{array}$$ (107)

$$\begin{array}{ccc} & & D_{v}V^{\prime }-D_{m}\frac{V}{m}m^{\prime }=h_{v}\left(V_{\infty }-V\right)|{}_{\hat{z}=100} \end{array}$$ (108)

which follow from (88)–(90). The oxygen concentration is fixed at *c* = 1 when $\hat{z}=100$ (chosen arbitrarily). We impose an additional boundary condition which fixes the wave speed *u* at $\hat{z}=100$, given by *u* = *h*_*w*_(*w*_∞_ + *m* − 1). This extra boundary condition allows us to use the bifurcation and continuation AUTO software [31] to solve (98)–(103) to calculate the wave speed, u.

### 3.8 Travelling wave solutions of spheroid growth

**Fig 9:**
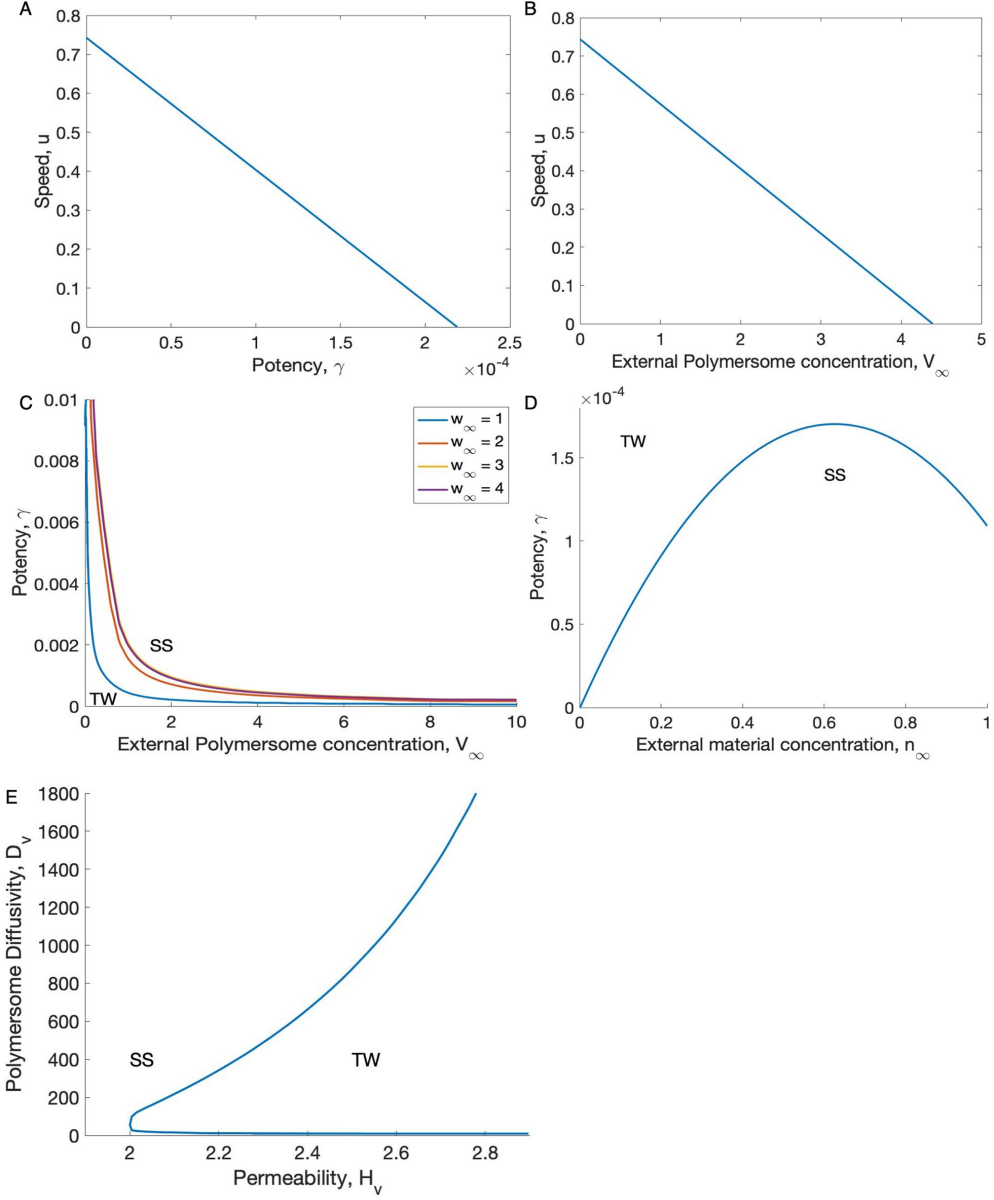
Travelling wave and steady state solutions for the tumour spheroid model. **(A)** The travelling wave velocity, *u*, with varying potentcy, *γ*, and fixed polymersome concentration at the spheroid boundary, V_∞_ = 2, and **(B)** travelling wave velocity with varying V_∞_ and fixed *γ* = 1 × 10^−4^. (*Middle and bottom*) Travelling wave: steady state bifurcation curves. (*Middle row*) Bifurcation curves for *γ* with **(C)** various external polymersome concentrations, *V*_∞_ and fixed external nutrient concentration *w*_∞_ = 1 ⋯ 5, and **(D)** with various cellular material concentrations, *w*_∞_, and a constant polymersome concentration, V_∞_ = 5. In **(E)** we see the bifurcation in the polymersome diffusivity and spheroid permeability parameter space. The rest of the parameter values used are give in Tables 1 and 2.

Fig 9 shows the wavespeed, u, against potency, *γ*, and external polymersome concentration, V_∞_. We can see that by increasing the potency of the polymersomes, *γ*, the wavespeed decreases to zero (A), indicating a bifurcation from travelling waves (linear growth) to steady-state where tumour growth is confined. As V_∞_ increases we observe similar dynamics, i.e. a greater concentration of polymersomes results in a more effective treatment (B).

In Fig 9, we also show the travelling wave:steady-state bifurcation in (V_∞_,*γ*) parameter space, with a varying external cellular material concentration, w_∞_ (C). We can see that, with increasing concentration of applied polymersomes, a lower polymersome potency is required for steady state solutions. We would expect that by increasing w_∞_ the tumour growth would increase resulting in a larger parameter region for travelling waves. We have found that this is true but only to a certain threshold value of w_∞_, (D).

In Fig 9(E) we explore the travelling wave:steady-state bifurcation in relation to the diffusion of the polymersomes and the permeability across the spheroid boundary. We notice that for a fixed value of h_*v*_ (for example, if h_*v*_ = 2.2) the solutions can be either travelling waves for very small or very large values of D_*v*_, but steady state solutions occur for intermediate values of D_*v*_.

**Fig 10:**
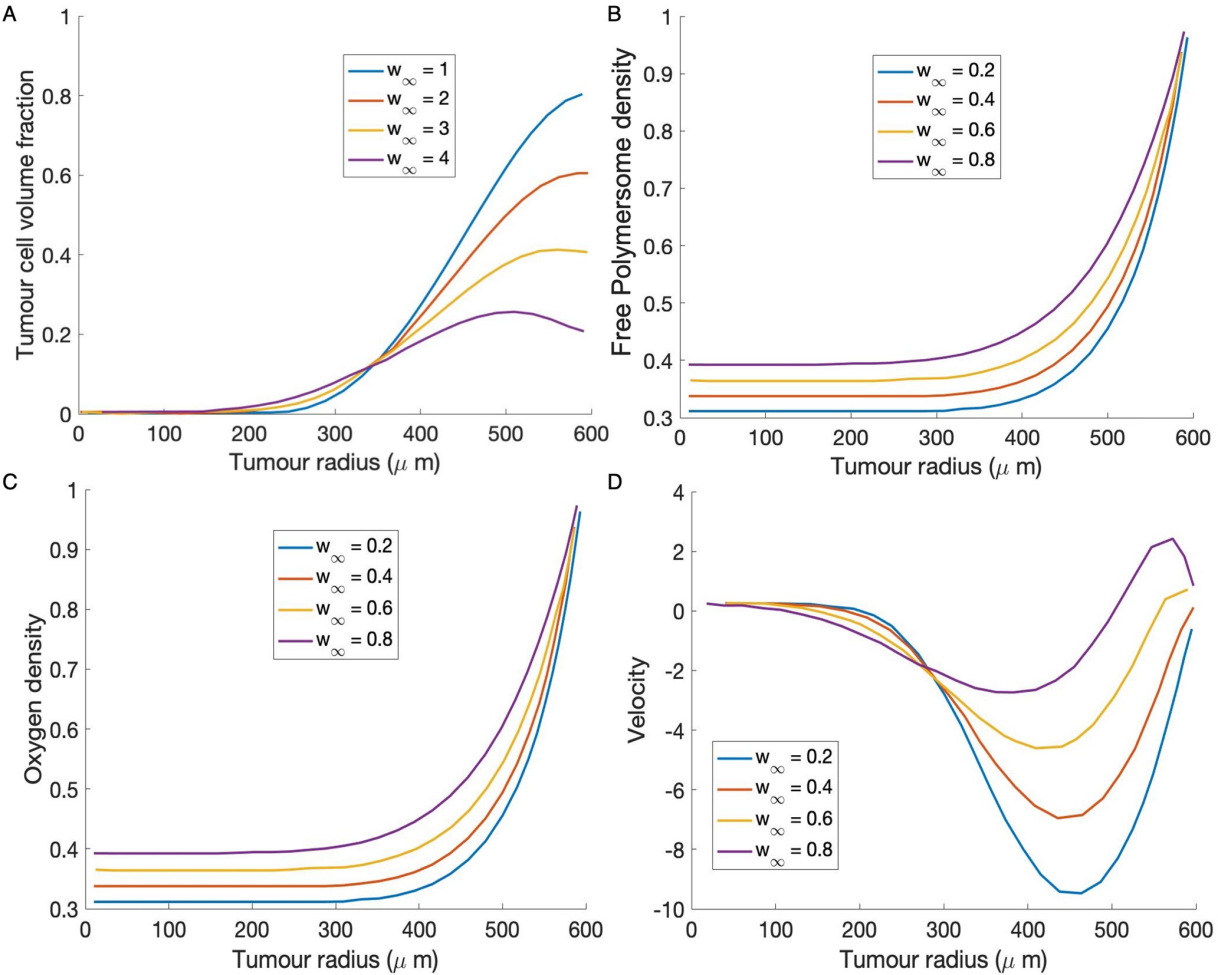
In **(A, B)** and **(C)** are the solution profiles for tumour cells, free polymersomes and oxygen, respectively, at varying values of external nutrient concentration, w_∞_ = 0.2 (blue),0.4,0.6,0.8 (purple). **(D)** Advection velocity with w_∞_ = 0.2, 0.4, 0.6, 0.8. We see a positive velocity at the tumour boundary with larger values of w_∞_. The rest of the parameter values used are give in Tables 1 and 2.

Finally, we examine the relationship between *γ*, *V*_∞_ and *w*_∞_ further by fixing V_∞_ and following the travelling wave, steady state bifurcation in (w_∞_,*γ*) parameter space. In Fig 10, we examine the behaviour of the tumour cells and the internal velocity field within the tumour spheroid for these parameter values. Typically the advection velocity within the spheroid is negative, which means that material is advected into the centre of the spheroid. However, when increasing w_∞_ over a certain threshold, we see that the advection velocity becomes positive near the spheroid boundary, this change in the direction of the velocity results in the polymersomes being kept at the tumour boundary where the tumour density is highest. As a consequence more viable cells are being targeted and subsequently a lower polymersome potency (*γ*) is required to give the same reduction in tumour size. We also show how the free polymersome and oxygen density vary over the spheroid radius for various value of *w*_∞_.

## 4 Discussion

A mathematical model for nanoparticle uptake via receptor-mediated endocytosis has been presented. The model is applied to two scenarios representative of relevant experimental designs for tumour therapy development. Firstly, the model was applied to cells in a well-mixed system, representative of 2D monolayer cells and polymersomes in a tissue culture flask, and secondly to investigate dynamics of growth in a tumour spheroid during and after polymersome application. For both approaches the model is nondimensionalised in order to evaluate the dominant features. The well-mixed system was investigated first which allowed spatial effects to be neglected, with a focus on the binding kinetics of the model.

The nondimensionalisation of the well-mixed model indicated that polymersomes are internalised after forming one bond with the cell, since the internalisation rate is several orders of magnitude higher than the binding rate (see Table 1). Based on this we made the assumption that the maximum number of bonds is low throughout the rest of the investigation. In the singular perturbation analysis, a single binding event is assumed and the system is further simplified by neglecting the polymersome dissociation rate, which is small compared to uptake, and linearising the functions that describe receptor recycling and cell death. These changes did not affect the observed early time system dynamics.

By carrying out this analysis some important biological insights regarding the early kinetics of the polymersome uptake are discovered. It was found that the maximum value for internalised polymersomes corresponds to a particular value of ligands approximately 500 (see Fig 5 which is consistent with the literature [21]. Hence this reinforces the hypothesis that there is an optimal number of ligands per polymersome. The findings from the perturbation analysis can be extremely useful for biologists working on nanoparticle targeted therapy as the number to targeted ligands on polymersomes is specific to the polymersome structure which can be modified during the manufacturing of the polymersomes. This approach could easily be applied to other types of nanoparticle to guide experimental design.

The findings from the well-mixed system were used in the spheroid model. In the well-mixed system internalisation happens at a low number of bonds which meant that during the simulations the maximum number of bonds, could be capped at a low number, reducing the required mathematical work load.

The spheroid consists of two phases, tumour cells and cellular materials, similar to the approach of the seminal work by Ward and King [22] and Byrne et al [32]. The novelty of this approach is the use of the spheroid model to understand polymersome uptake and distribution in a system which mimics polymersome delivery from a source. The growth of the spheroid depends on available nutrients which diffuse across the spheroid boundary. A quasi-steady state assumption is made for bound polymersomes. Although the spheroid does not have vasculature, by placing the nanoparticles on the cell surface we can understand how cells will be affected at varying distances from a nanoparticle source, for example a blood vessel, by looking at the gradient from the polymersome source at the tumour surface to the cells towards the centre of the spheroid.

From Table 2 we see that the diffusion coefficient for oxygen is an order of magnitude higher than that of the polymersomes, since oxygen is a much smaller molecule. This implies that oxygen should diffuse further into the spheroids than the polymersomes over a given time period meaning some cells may able to proliferate that aren’t reached by the polymersomes. After application of polymersomes to the spheroid surface it was found that the highest concentration of free polymersomes occurs at the boundary. As the spheroid shrunk due to cell kills by polymersomes, the polymersomes moved closer to the spheroid centre due to the reduction in spheroid radius. At all time points during the polymersome application the polymersomes remain close to the surface. This is likely to be because there is a high density of proliferating tumour cells and this density poses a barrier to diffusion. The same is also seen for oxygen, albeit oxygen does penetrate further into the spheroid. The limitation of oxygen to the spheroid periphery results in a necrotic core which arises naturally from our simulations and is in agreement with experimental findings [16, 18, 33, 34].

Two internalisation functions are considered for multiple binding events. A constant function is considered, which was also used in the well-mixed system, as well as a more biologically realistic internalisation function, in which a polymersome must be bound by a given number of complexes before internalisation can occur. The bond dependent function mimics the membrane deformation that occurs during receptor-mediated endocytosis. We introduce an approximation to the free receptor concentration as the multiple binding framework becomes algebraically intractable to solve when the maximum number of bonds that can form is greater than 2.

Numerical simulations indicate that the spheroid radius is dependent on the internalisation function. The bond dependent function requires the polymersomes to reach the value of the activator before being internalised. For values of the activator below 5, the predictions of the constant and bond dependent functions match. However, as the activator is increased to 5 the polymersomes do not impact the growth of the spheroid, indicating a threshold value exists for effective treatment over the time period tested. Therefore, we conclude that internalisation function plays a critical role in treatment success and effects model predictions.

For the case of the bond dependent internalisation function we found that the number of bonds required before internalisation had a short term effect on the tumour radius, but over long time periods the solutions were the same. Due to this result, if multiple polymersome applications were to be considered, it can be hypothesised that the time between administration could result in either a decrease in spheroid size or no effect, depending on the value of this threshold.

Various nutrient concentrations in the space surrounding the spheroid were explored. It was found that, up to a threshold, with increasing nutrient concentration there was an increase in growth and more potent polymersomes were to attain saturated growth. After the threshold the model predicts that we are more likely to see saturation of growth when using less potent polymersomes. In this case, the internal velocity within the spheroid came into play. By increasing the nutrient concentration past the threshold, the gradient of cellular material switched direction, creating an opposite internal velocity, causing the polymersomes remained close to the spheroid edge. This indicates that the nutrient concentration in the media should be considered when designing experiments.

The influence of permeability of the spheroid surface and the polymersome diffusion rate on spheroid growth was also explored. It was found that for certain values of permeability spheroid growth or saturation was possible, although a lower permeability generally resulted in steady-state solutions. For very small or very large values of the polyermsome diffusivity, travelling waves occur whereas intermediate values result in growth saturation. This implies that nanoparticles should be designed with intermediate diffusion coefficient and a high permeability to the spheroid boundary in order to restrict spheroid growth.

It should be noted that a number of key assumptions were made, and parameter values were estimated or drawn from similar, but not the same models and data sets were used in order to achieve these results. Therefore, the results are valid for the parameter set we have used, but this parameter set may be subject to change once further experimental data can be obtained which could influence the system dynamics. For example a number of simplifying assumptions were made based on the size of some parameters, such as the dissociation constant was assumed to be negligible. Likewise, the numerical solutions presented in section 3.4 for the spheroid model make a number of assumptions about the quasi-state regimes. If these parameters and assumptions are found to be significantly different from the ones we have used this could have significant impact on the model predictions and analytical tractability of the model.

However, these assumptions and parameter choices were made due to the lack of available experimental data on which to base the work, and in fact the lack of experimental data is one of the key motivations for this work. The processes which are modelled in this paper, particularly of cellular uptake of polyermsomes, and subsequent release of chemotherapy drug, is extremely difficult, if not impossible to image for each part of the process given current technology and so mathematical modelling offers an alternative to this.

Despite the difficulty in finding data to parameterise the model, the results still offer key insights into the system dynamics and has highlighted which parameters are important to know accurately. The use of two different systems, one reflective of a 2D monolayer and one of 3D tumour spheroid, has allowed various characteristics of polymersome uptake to be elucidated, particularly over various time periods with respect to uptake of polymersomes by cells, as well as allowing the use of parameter values predicted in the well-mixed system to be used in the spheroid model. The agreement of the models on certain kinetics strengthens the conclusions that can be made. Both approaches revealed that most polymersomes are internalised after only a few bonds are made between the polymersome and cell.

## 5 Conclusion

We have demonstrated the application of a new model of cellular uptake of nanoparticles, for example polymersomes, via receptor-mediated endocytosis that is parameterised with experimental data to investigate uptake in tumour spheroids. We make predictions about nanoparticle design, namely the number of ligands on the surface and diffusion and permeability coefficients, which can be fed into future experimental work. We have also shown that, over long time periods, spheroid growth is independent of polymersome internalisation function and that polymersome distribution is limited to the outer edge of the spheroid, close to their source. Future work will see further development of the mathematical model and applications to other systems.

## Supporting information

S1 File (PDF)
