# Supplementary material for: A mathematical investigation into the uptake kinetics of nanoparticles in vitro
Source: PLoS One. 2021 Jul 22;16(7):e0254208. doi: 10.1371/journal.pone.0254208 (PMC8297806; doi:10.1371/journal.pone.0254208)
Supplement: S1 File — (PDF) [file pone.0254208.s001.pdf]

S1 File

1.1 Parameterisation

Without any specific data available, we assume that the decay rate of the receptors on the cell surface is $d_f = 0.03$ min^{-1} [26]. We derive the production of cell receptors, c_1, by assuming that, when there is no binding, production must equal decay, so that $c_1 = d_f$,

The parameters c_2 and c_3 characterise the Hill function for receptor recycling. For simplicity we make $c_2 = c_1$. The parameter c_3 is value at which the half maximum of the function $R(b_{tot})$ is reached. We assume that this value is $c_3 = 0.5$ mol cell^{-1} and we choose the exponent, α, is equal to one because the binding rate is constant, i,e, not dependent on the number of bonds formed.

We let the proportions ρ_f, ρ_l vary within $(0, 1]$. Using Ghaghada *et al* [21] as a guide, we further assume that there are initially 10^4 receptors per cell. We assume that there are 10^{10} polymersomes per cm^3 and 5×10^7 cells per cm^3 [25], with the tumour cells being at carrying capacity at $t = 0$ of the simulations. We assume that tumour cells

divide roughly once per day, therefore the growth rate, r, is given by $r = 1$ day^{-1} which has been used widely in previous mathematical models for tumour growth (see e.g. [29]).

From Ahmed *et al* [8], we take the half-life cytotoxicity from the polymersomes containing taxol to be roughly 10 hours. Therefore, we use this as our initial estimate for the death rate of tumour cells μ, i.e. $\mu = \frac{\ln 2}{10}\text{h}^{-1} = 0.0012$ min^{-1}. From data provided by Prof Craig Murdoch from the School of Clinical Dentistry in the University of Sheffield, we notice that the time-frame of tumour size reduction occurs much faster than the half-life of 10 hours, hence we increase the order of magnitude of μ to suit and let $\mu = 1.2\text{min}^{-1}$ to make the numerical solutions match the time-frame of the experiments.

It is known that the half-life of the free drug taxol is roughly 10 hours, similar to the half-life cytotoxicity of the polymersomes. We assume that the drug half-life is the time it takes for half the drug to be used by the cells, and so we take the same value for the decay rate of the drug, d_p, as that for μ. From Ahmed *et al* [8], we see that the half-life for polymersomes to leave endosomes is roughly 5 hours. Hence the rate of polymersome rupture, d_b can be calculated by $d_b = \frac{\ln 2}{5}\text{h}^{-1} = 0.0023\text{min}^{-1}$. As with μ, however, we found this value to give unrealistic results as compared to experiments. We found a much better match when we take $d_b = 2.3\text{min}^{-1}$ which corresponds to a half life of 18 hours.

Although every effort was made to parameterise the model using well established data from the literature, relevant data was sparse and thus a similar model of liposomes was used to find most of the parameter values. Despite using a similar model there are a few remaining unknown parameters which are estimated to match experimental observations. The parameters along with the appropriate units for this model, are shown in Table 1. To parameterise our model we found values in literature, specifically in Ghaghada *et al* [21] due to the similarities in the binding kinetics.
